# Doping Engineering of Single-Walled Carbon Nanotubes by Nitrogen Compounds Using Basicity and Alignment

**DOI:** 10.1021/acsami.2c00970

**Published:** 2022-05-18

**Authors:** Bogumiła Kumanek, Karolina Z. Milowska, Łukasz Przypis, Grzegorz Stando, Karolina Matuszek, Douglas MacFarlane, Mike C. Payne, Dawid Janas

**Affiliations:** † Department of Organic Chemistry, Bioorganic Chemistry and Biotechnology, Silesian University of Technology, B. Krzywoustego 4, 44-100 Gliwice, Poland; ‡ TCM Group, Cavendish Laboratory, University of Cambridge, 19 JJ Thomson Avenue, Cambridge CB3 0HE, United Kingdom; § CIC nanoGUNE, Tolosa Hiribidea 76, 20018 Donostia-San Sebastián, Spain; ∥ Ikerbasque, Basque Foundation for Science, 48013 Bilbao, Spain; ⊥ Monash University, School of Chemistry, Clayton, VIC 3800, Australia

**Keywords:** carbon nanotubes, electrical properties, thermoelectric properties, doping, nitrogen compounds

## Abstract

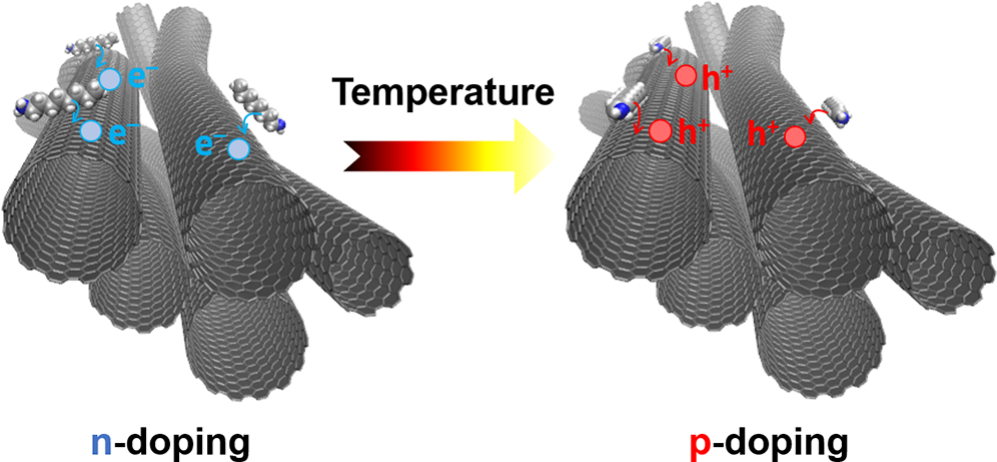

Charge transport properties in single-walled carbon nanotubes (SWCNTs) can be significantly modified through doping, tuning their electrical and thermoelectric properties. In our study, we used more than 40 nitrogen-bearing compounds as dopants and determined their impact on the material’s electrical conductivity. The application of nitrogen compounds of diverse structures and electronic configurations enabled us to determine how the dopant nature affects the SWCNTs. The results reveal that the impact of these dopants can often be anticipated by considering their Hammett’s constants and p*K*_a_ values. Furthermore, the empirical observations supported by first-principles calculations indicate that the doping level can be tuned not only by changing the type and the concentration of dopants but also by varying the orientation of nitrogen compounds around SWCNTs.

## 1 Introduction

One of the key problems of the modern world is the inefficient utilization of the limited amount of resources that we have at our disposal. Civilization is highly dependent on energy sources such as fossil fuels,^1^ which have sustained our growth for several centuries. However, since these feeds cannot replenish themselves, we will inevitably run out of them one day. In the 1950s, the concept of utilization of renewable resources emerged, and its share of global primary energy consumption has risen steadily over the years to alleviate the highlighted issue.^2^ The use of green energy as an alternative may also minimize the problem of global warming. Unfortunately, the contribution of energy generated from sustainable sources is currently only ca. 4%, so other measures should be implemented in the meantime. One of the possibilities is to increase the overall energy efficiency of the devices and processes surrounding us. There is much to gain in this area because about 2/3 of generated energy is currently wasted as heat.^3^ Therefore, facile strategies for how to recover and reuse thermal energy are pressing.

Thermoelectric devices are capable of transforming waste heat into valuable electrical energy. Carbon nanotubes (CNTs) and single-walled CNTs (SWCNTs), in particular, are promising because they offer appreciable performance.^4^ At the same time, they can be made from renewable resources.^5^ A thermogenerator should simultaneously have high electrical conductivity, high Seebeck coefficient, and low thermal conductivity to attain the best performance. The measures that quantify a given material’s suitability for thermoelectric properties are the power factor (PF) and the figure of merit. The former disregards the impact of thermal conductivity, which is often challenging to determine at elevated temperatures, so PF values are routinely reported.^6^ The PF is defined as PF = γ*S*^2^, where γ and *S* indicate the electrical conductivity [S/m] and Seebeck coefficient [μV/K], respectively^3^ (γ is used in this work as the symbol denoting electrical conductivity to avoid confusion with Hammett substituent constants discussed later, both of which are commonly denoted by σ). The problem in achieving the best possible PF values is related to the fact that electrical conductivity is proportional to carrier density, while the Seebeck coefficient exhibits the inverse relation.^3^ Therefore, to obtain the best possible thermoelectric parameters, it is necessary to find trade-off conditions.

For instance, this is possible through doping, which can appropriately modify the electronic properties of SWCNTs by optimizing the Fermi level.^7,8^ Such doping can be executed in various ways. The doping atoms can be incorporated into the graphitic lattice,^9−11^ intercalated within the inner cavity,^12,13^ or adsorbed on the surface of SWCNTs.^8,11^ Nanoguchi et al. tested various electron-donating/-withdrawing compounds as doping agents.^14^ The highest absolute value of the Seebeck coefficient they obtained was ca. 80 μV/K for carbazole- and triphenylphosphine-doped SWCNTs. Furthermore, the best PF value of 26 μW/mK^2^ was recorded for tetracyanoquinodimethane (TCNQ). This chemical compound contained four nitrile groups, so one could deduce that nitrogen played a crucial role in doping the material. Moreover, Ruy et al. demonstrated that SWCNTs after doping with *N*-methyl-2-pyrrolidone were characterized by a large Seebeck coefficient and power factor of 60 μV/K and 72 μW/mK^2^, respectively.^15^ Other results confirming the positive effect of nitrogen compounds were presented by Wu et al.^16^ When SWCNTs were combined with naphthalene diimide or perylene diimide, the material exhibited Seebeck coefficient values of −60.2 μV/K and −52.4 μV/K and PF values as high as 135 μW/mK^2^ and 112 μW/mK^2^, respectively. These outcomes indisputably showed that SWCNTs doped with nitrogen compounds render improved thermoelectric properties. It is evident that more focus should be given to this emerging area to understand the phenomena behind the positive influence of nitrogen on the electrical and thermoelectric properties of SWCNTs.

In our study, we have extensively analyzed the impact of various compounds containing nitrogen atoms on these attributes of SWCNT films. The study aimed to determine a possible correlation between the structural and electronic properties of the dopant used and the material’s final electrical and thermoelectric properties. The electrical conductivity of nitrogen-doped SWCNT films was studied as a function of the doping agents. The results reveal that the electrical conductivity of SWCNT films doped with nitrogen atoms can, in most cases, be predicted based on the Hammett substituent constants or p*K*_a_ values of the dopants. Furthermore, first-principles calculations highlight that thermoelectric properties of SWCNT films can be controlled not only by the proper selection of nitrogen dopants and tuning their concentrations but also by rearranging the orientation of nitrogen compounds around SWCNTs. Lastly, by taking octylamine-doped SWCNT films as an example, we explain the underlying mechanisms responsible for dramatic changes to the thermoelectric properties of the material caused by dopant rearrangement with temperature.

## 2 Results and Discussion

### 2.1 Electrical Conductivity at Room Temperature

**Figure 1:**
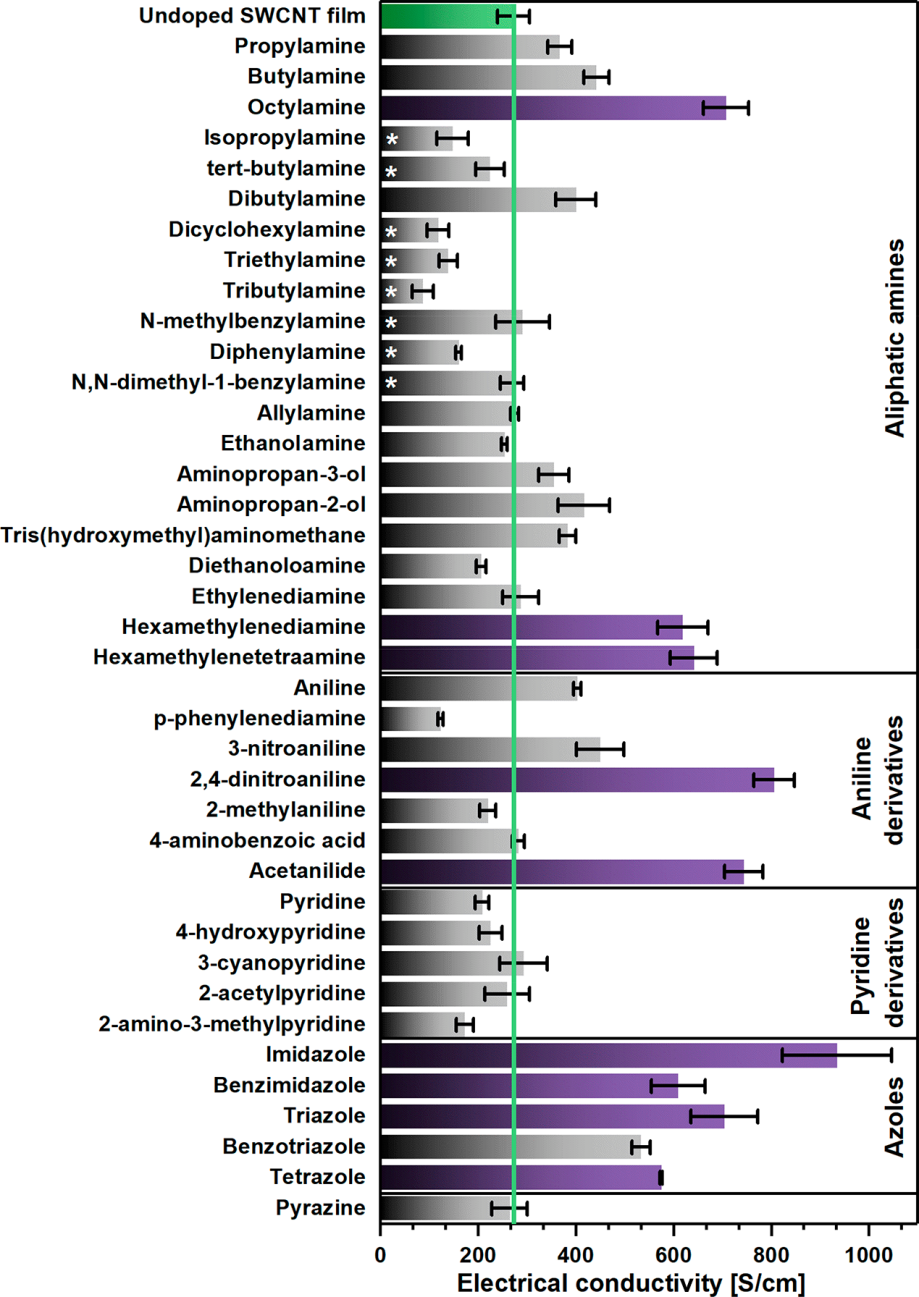
Electrical conductivity for nitrogen-doped SWCNT films. Aliphatic amines branched with hydrocarbon groups are indicated with an asterisk. The doping agents for which further more detailed analyses were performed are highlighted in purple.

We initiated the study by characterizing the electrical conductivity of SWCNT films doped with various nitrogen-bearing compounds at room temperature (Figure 1). The application of diverse amine compounds having various types/numbers of amino groups and substituent patterns allowed us to determine how the structure of the dopant molecule affects the values of electrical conductivity (vide infra). The chemical compounds explored were divided into four categories: aliphatic amines, aniline derivatives, pyridine derivatives, and azoles. For the best samples (marked in purple), for which the value of electrical conductivity exceeded that of the undoped material at least 2-fold, the electrical properties of the material were also evaluated as a function of temperature. The electrical conductivity of the reference (neat SWCNT film) amounted to 272 ± 32 S/cm, which agrees with earlier reports on the electrical conductivity of films made of the same SWCNT precursor.^17^

#### 2.1.1 Aliphatic Amines

**Figure 2:**
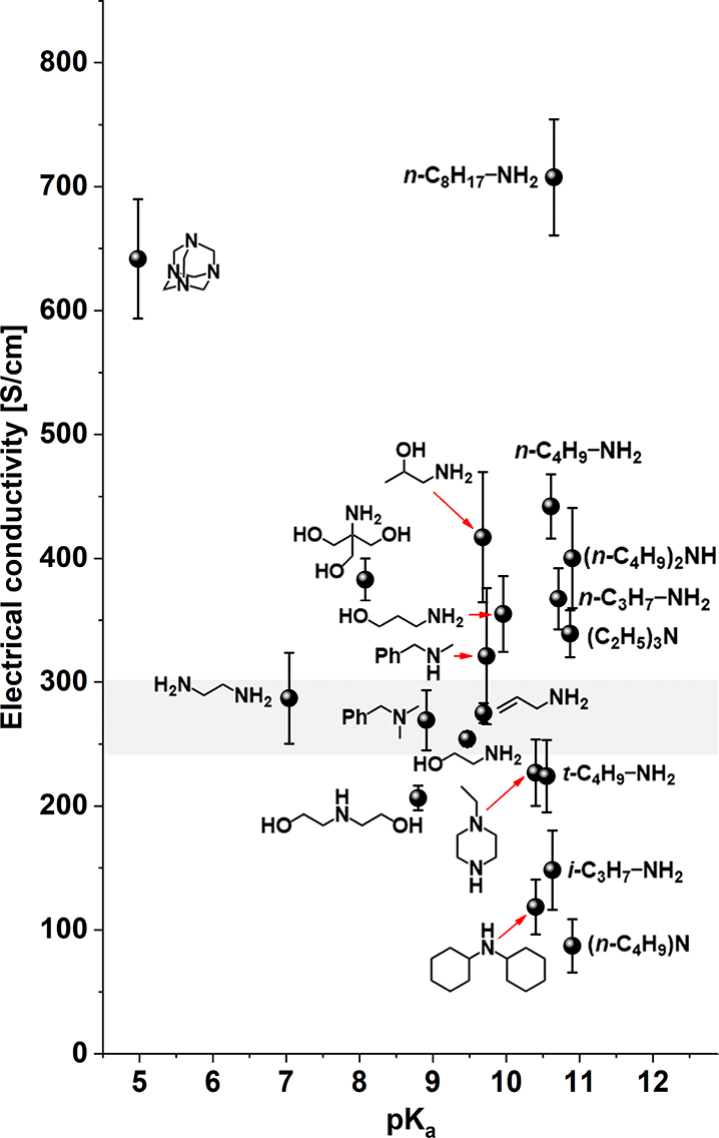
p*K*_a_ values for aliphatic amines correlated with the measured electrical conductivity of doped SWCNT films. The shaded area depicts the electrical conductivity of neat SWCNT films, which considers the statistical error.

For primary aliphatic amines, we observed that linear amines caused an increase in electrical conductivity in contrast to the branched ones (indicated with an asterisk in Figure 1). This relationship is illustrated well by the propylamine and isopropylamine pair (Figure 2). The addition of the former caused an increase in electrical conductivity to 367 ± 25 S/cm, whereas the latter decreased the conductivity of the SWCNT film to 148 ± 31 S/cm. This trend was also replicated by using longer primary amines such as butylamine or *tert*-butylamine, which reached electrical conductivities of 442 ± 26 S/cm and 224 ± 29 S/cm, respectively. Furthermore, when other branched secondary and tertiary amines were employed, e.g., dicyclohexylamine or tributylamine, they also caused deterioration of electrical conductivity down to 108 ± 22 S/cm and 87 ± 21 S/cm, respectively. The negative impact was especially noticeable in the case of tertiary amines (such as tributylamine), the incorporation of which produced SWCNT films of the worst electrical conductivity.

We hypothesize that two factors hamper the interaction between the nitrogen atoms of the dopant and the SWCNT sidewall, thereby limiting the enhancement of the electrical conductivity. First, from the structural point of view, it is likely that the doping species align on the SWCNT surface to maximize the contact with the alkyl chains or aryl substituents. Thus, these groups should face the SWCNTs to lower the system’s energy by establishing van der Waals interactions. Therefore, branching the aliphatic amines with alkyl or aryl groups places the nitrogen atoms further away from the SWCNT surface, impairing the charge transfer between the molecule and nanotube, which reduces the doping effect (see Table S1 and Figure S1). The phenomenon is most notable in tertiary amines. All three hydrocarbon substituents adsorb preferentially on the SWCNT surface, which keeps the nitrogen locus distant from the SWCNT due to the tetrahedral arrangement of atoms. In such a case, the alkyl chains on the surface can even be undesirable as they may impair the ability of the SWCNT network to propagate charge between individual SWCNTs and their bundles. We observed this phenomenon in several cases above. Second, steric hindrance in the branched amines does not enable efficient packing of the doping species on the SWCNT surface. Conversely, when these molecules have a linear form, they are much more likely to exhibit certain regioregularity patterns. Consequently, since more linear alkyl amine molecules can be deposited on the SWCNTs, the doping effect is intensified. This hypothesis is supported by calculations (Table S1 and Figure S1) and may explain why the best results were obtained for SWCNT networks doped with linear alkyl amines. It has to be noted that certain branched amines cause deterioration of the electrical conductivity of SWCNT films. This issue remains unresolved and will be the subject of future investigations.

Furthermore, another finding is that for primary amines the longer the alkyl chain, the larger the boost to the charge transport capabilities. For example, propylamine, butylamine, and octylamine reached electrical conductivity values of 367 ± 25 S/cm, 442 ± 26 S/cm, and 707.5 ± 47 S/cm, respectively. This outcome supports our conclusions mentioned above that it is essential to (i) ensure appropriate interaction of the dopant with the SWCNTs and (ii) ensure intimate contact of the nitrogen atoms with the SWCNT surface. Regarding the former, the longer the alkyl chain, the stronger the interaction of the host (SWCNT) with the guest (amines) is due to an increased number of formed van der Waals interactions. The practical implication of this phenomenon is that the doping species are not prone to desorption, so they are not removed during preparation or subsequent storage. Second, the primary alkyl amines have no spatial constraints, which could interfere with the interplay between nitrogen atoms and the SWCNTs.

We also observed that the electric conductivity increases slightly in the presence of substituents that affect the electron distribution in the dopant molecule. This effect is best seen in the propylamine and aminopropan-2-ol pair, for which the electrical conductivity was 367 ± 25 S/cm and 417 ± 52 S/cm, respectively. The presence of the hydroxyl group increased the electrical conductivity by 13% more above that of the unsubstituted compound due to the electron-donating character of this group. Interestingly, to reach such an enhancement, the hydroxyl derivative of the primary alkyl amine has to have a sufficiently long alkyl chain. This is demonstrated well by ethanolamine, which contains the hydroxyl group, but the alkyl chain is too short to obtain a favorable interaction between SWCNTs and the doping agent.

Lastly, we wanted to validate whether the basicity of the aliphatic amine dopants can be used to predict the impact on the electrical conductivity of SWCNT films. However, no obvious correlation could be established between the electrical conductivity of doped SWCNT films and the corresponding p*K*_a_ values of the dopants. This result was consistent with the conclusions drawn above that the impact of the doping agent on the SWCNT films in the case of aliphatic amines is dictated by the structural compatibility between the host and the guest due to the effects indicated previously.

#### 2.1.2 Aniline Derivatives

To study the nitrogen doping of SWCNTs in greater detail, we moved on to aromatic amines. Aniline derivatives are chemical compounds that are convenient for use as doping species. Due to their compatibility with SWCNTs and structural diversity, they can be employed to study various relations. In the case of aromatic compounds, it is more appropriate to consider the Hammett substituent constants, which take into account both the basicity of the molecule as well as the inductive effects.

**Figure 3:**
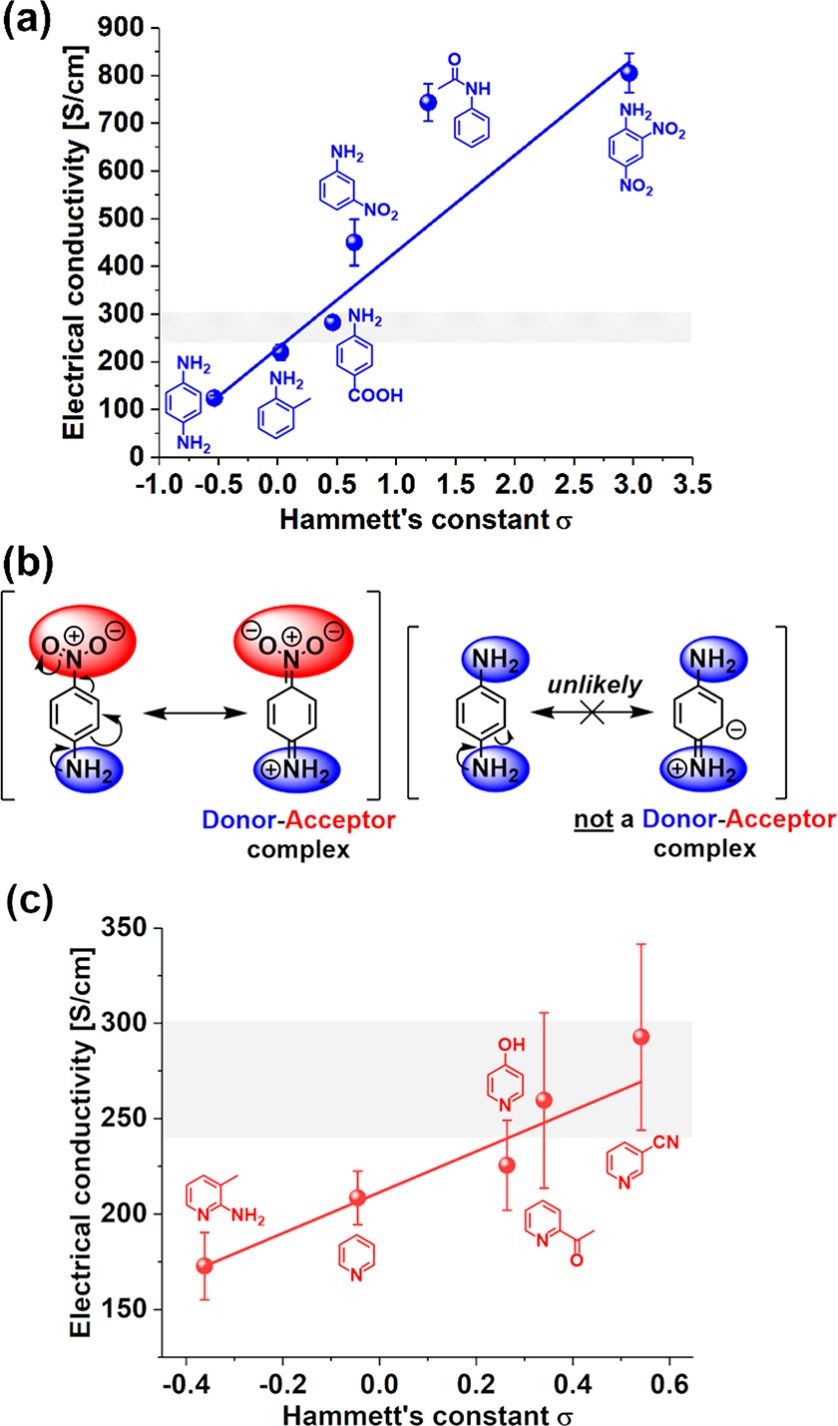
(a) Impact of the substituent in anilines on the Hammett substituent constants and its correlation with the measured electrical conductivity of doped SWCNT films. (b) The mechanism of the formation of the donor–acceptor complex. (c) Impact of the substituent in pyridines on the Hammett substituent constant and its correlation with the measured electrical conductivity of doped SWCNT films. The shaded area depicts the electrical conductivity of neat SWCNT films, which considers the statistical error.

Interestingly, we observed a linear relationship between the value of the Hammett substituent constant and the electrical conductivity of the SWCNT films doped with anilines (Figure 3A). The conductivity increased proportionally to the value of the Hammett substituent constant, suggesting that the positive charge in the benzene ring is favorable for enhancing the electrical conductivity. For instance, electron-rich *p*-phenylenediamine gave SWCNT films of poor electrical conductivity of 123 ± 5 S/cm, whereas electron-poor 2,4-dinitroaniline enhanced the electrical conductivity up to 805 ± 42 S/cm. Likely, the exceptional improvement of the electrical conductivity in this case of the latter dopant stems from the fact that this compound can form a so-called donor–acceptor complex^18^ (Figure 3B). The presence of electron-donating and electron-withdrawing groups on the benzene ring facilitates a push–pull effect, stabilizing the positive charge. This phenomenon intensifies the ability of the dopant to extract electrons from SWCNTs, which in turn makes the p-doping effect stronger. Moreover, since this compound possesses two nitro groups, both of which are in the appropriate positions to accommodate negative charge, 2,4-dinitroaniline can strongly dope the SWCNTs. On the other hand, in the case of electron-rich *p*-phenylenediamine, such a complex cannot be formed, so the SWCNT films cannot be effectively doped with this chemical compound. Actually, the addition of *p*-phenylenediamine deteriorated the electrical conductivity of the film, supposedly by interfering with the charge propagation between the individual SWCNTs.

Another phenomenon should also be highlighted at this point. We found that 4-aminobenzoic acid cannot facilitate the enhancement of the electrical conductivity in contrast to 3-nitroaniline. Although these compounds have a similar Hammett’s constant, the inductive effect of the nitro group in the meta position is stronger than that of the carboxylic group in the para position. Therefore, the latter dopant is more electron deficient, so it can withdraw electrons from SWCNTs more effectively, thereby enhancing their electrical conductivity.

#### 2.1.3 Pyridine Derivatives

To study the role of nitrogen in greater detail, we studied a class of compounds commonly referred to as pyridines, which contain the nitrogen atom in the aromatic ring. In this case, we observed a trend similar to that for anilines (Figure 3C). The higher the Hammett substituent constant, the stronger the p-doping effect. The most significant increase in electrical conductivity was obtained for 3-cyanopyridine, reaching 293 ± 49 S/cm. It is evident that the enhancement was much smaller than when anilines were employed as dopants. However, considering the Hammett substituent constants of both classes of chemical compounds, these results are coherent. The most electron-withdrawing pyridine compound (3-cyanopyridine) had a Hammett substituent constant of merely 0.54, while for 2,4-dinitroaniline, it was as high as 2.97. The much lower value of this parameter for pyridines results from the fact that the nitrogen atom present in the aromatic ring donates electrons to the system, which, as we observed, is unwelcome for SWCNT doping. The aromatic ring of the nitrogen dopant should ideally have low electron density to make SWCNTs the most conductive.

**Figure 4:**
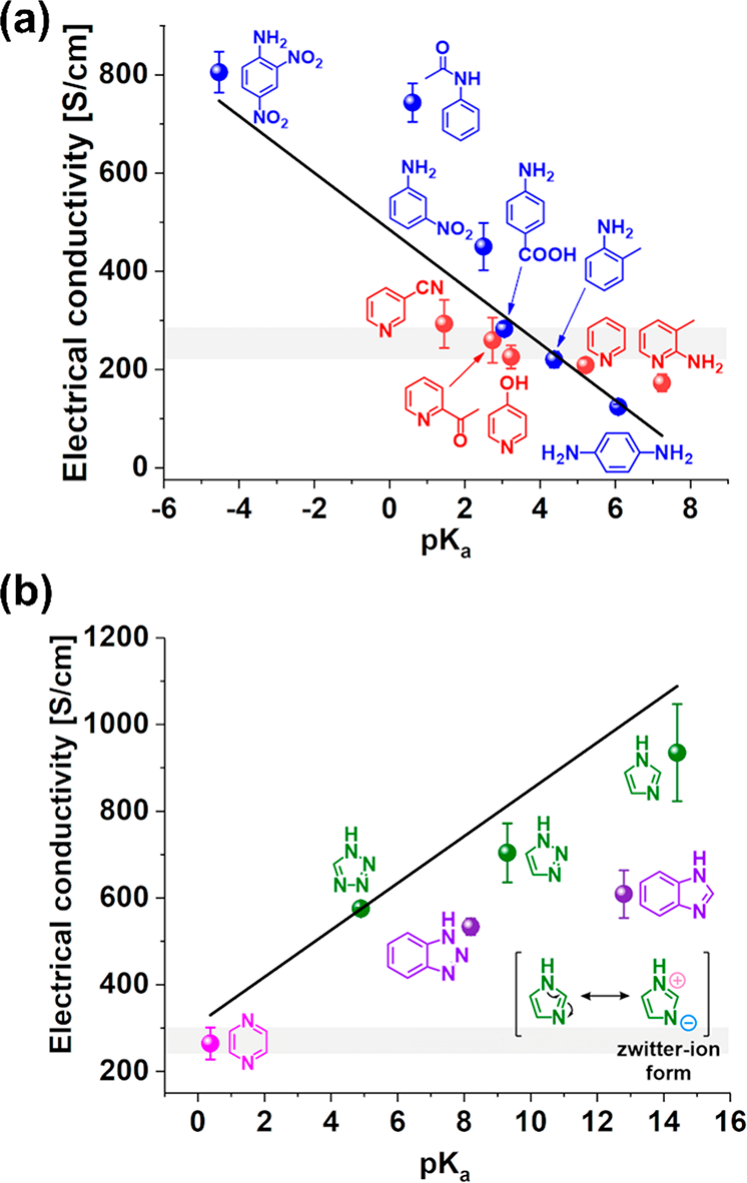
(a) p*K*_a_ values for derivatives of aniline and pyridine as well as (b) pyrazine and azoles and their correlation with the measured electrical conductivity of doped SWCNT films. The shaded area depicts the electrical conductivity of neat SWCNT films, which considers the statistical error.

At this point, we decided to inspect whether the enhancement of electrical conductivity could be directly related to the basicity of the doping agents. A clear relation between the p*K*_a_ values and electrical conductivity was observed for both anilines and pyridines (Figure 4A). The less basic the dopant (low p*K*_a_), the stronger the increase in electrical conductivity, which resonates well with the previous discovery that electron-poor nitrogen compounds exhibit the highest doping performance. Furthermore, there is a correlation between p*K*_a_ of the dopant and electrical conductivity of the doped SWCNT films for anilines and pyridines in contrast to aliphatic amines (Figure 2). This finding supports the previously mentioned hypothesis that short or branched aliphatic amines cannot effectively deposit on the SWCNT sidewall. In contrast, the planar core of the aromatic ring in anilines and pyridines facilitates proper adsorption. The flat aromatic or heteroaromatic rings of anilines or pyridines, respectively, in addition to van der Waals forces, interact with the SWCNTs by π–π stacking, which promotes the modulation of the charge transport characteristics in a predictable way. Conversely, since the interactions between the nitrogen atoms of the aliphatic amines and the SWCNTs are ineffective, analogous correlation cannot be elucidated.

#### 2.1.4 Azoles and Pyrazine

Lastly, we wanted to determine the impact on the electrical conductivity of SWCNT films of N-heterocyclic aromatics containing more than one nitrogen atom (Figure 4B) in the ring. For these experiments, we selected pyrazine and azoles. Since these chemicals had no substituents, we used their p*K*_a_ values to gauge the role of their structure on the electrical properties of the SWCNTs. In this case, we discovered a trend opposite to that for anilines and pyridines (Figure 4A). The dopants with higher p*K*_a_ values demonstrated better conductivity. The worst results were obtained for pyrazine (p*K*_a_ of 0.37), for which electrical conductivity amounted to 264 ± 37 S/cm, while the highest conductivity value, i.e., 935 ± 112 S/cm, was reached by imidazole (p*K*_a_ of 14.4). Pyrazine has two sp^2^ nitrogen atoms in its structure, whereas imidazole has one sp^3^ and one sp^2^ nitrogen atom. In general, nitrogen with sp^2^ hybridization has a greater affinity to act as a charge acceptor, while sp^3^ nitrogen behaves as a charge donor. As a consequence, imidazole can exhibit a zwitterionic resonance structure, which delivers a positive charge to the aromatic ring. The zwitterion structure resembles the donor–acceptor complex mentioned in the case of anilines capable of causing the push–pull effect. This phenomenon gives rise to depletion of electron density on the aromatic ring, which holds the key to the notable enhancement of electrical conductivity of SWCNTs. On the other hand, in the case of pyrazine, such a structure is not possible, so the electrical conductivity of SWCNTs remains at the same level.

Triazole and tetrazole gave effects that were between those of imidazole and pyrazine. These azoles have additional sp^2^ nitrogen, which shifts the properties to those observed for pyrazines. Although creating the desired donor–acceptor complex is still possible in their case, the appropriate electron density in the aryl ring cannot be established. Hence, the electrical conductivity increases only to 704 ± 68 S/cm and 575 ± 2.5 S/cm for triazole and tetrazole, respectively.

To test the hypothesis that electron deficiency in the aromatic ring of the nitrogen doping compound is essential for improving the electrical conductivity of SWCNT films, analogous electrical conductivity measurements were also performed for electron-rich benzotriazole and benzimidazole. Their incorporation in SWCNT films gave poor electrical conductivity values of 534 ± 19 S/cm and 609 ± 55 S/cm, respectively. The presence of a benzene ring increases the molecules’ electron density (compared with simple triazole and imidazole), which deteriorates the electrical conductivity of the SWCNT films, thereby validating the postulated theory.

### 2.2 Structure and Thermal Stability

For doping to be successful, it has to enhance the electrical properties of the material and should not affect its structure or the thermal stability. To verify that it is indeed the case, we first employed Raman spectroscopy for the selected group of dopants which gave at least a 2-fold improvement to the electrical conductivity of SWCNT films (Figure S2). The most common approach to judge whether the nanocarbon was affected by the processing is to determine the ratio of intensities of the disorder band D to that of the graphitic lattice G. These features reveal the abundance of sp^3^ and sp^2^ carbon atoms, respectively. The starting material was of high quality, as the *I*_D_/*I*_G_ amounted to only 0.014. The addition of all of the selected dopants did not affect the crystallinity. The observed deviations at this level can be ascribed to uncertainty in the determination of this property. Even after adding acetanilide, the value of *I*_D_/*I*_G_ was 0.021, i.e., still very low.

Next, we studied the stability of the doping as a function of temperature. For this purpose, thermogravimetric analysis was carried out for the same selected SWCNT films (Figure S3). Upon doping, the thermal characteristics of SWCNT networks changed only by a small amount. The temperature of the maximum rate of degradation of the parent material of 681 °C shifted to 642–774 °C, depending on the dopant type. We also observed the appearance of signals, indicating weight loss at lower temperatures corresponding to the removal of the doping agent, but these emerge only above 100 °C (Table S2). In most cases, these temperatures overlap with boiling point or projected decomposition temperature of the corresponding chemical compounds. Example thermograms are presented in Figures S4 and S5.

To verify how a prolonged exposure of the doped SWCNT material affects the thermal stability in the operational conditions, we decided to evaluate these materials as thermogenerators within the temperature range of rt up to 100 °C. Once again, samples of the dopants that caused at least 200% enhancement to electrical conductivity at room temperature were subjected to such analysis.

### 2.3 Electrical and Thermoelectric Properties at Elevated Temperatures

**Figure 5:**
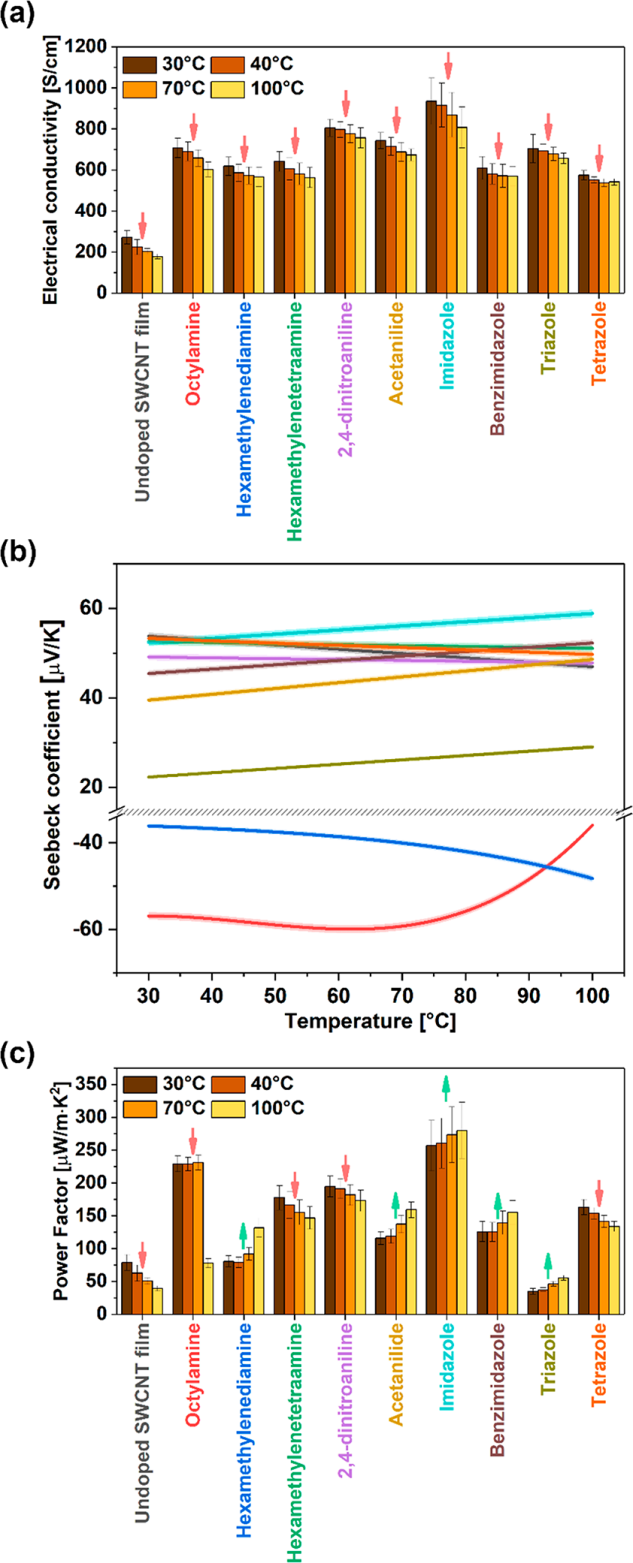
(a) Electrical conductivity, (b) Seebeck coefficient, and (c) power factor values from room temperature up to 100 °C for doped SWCNT films. Colors of the curves in panel (b) correspond to the descriptors on the *x*-axis of panels (a) and (c).

Measurement of electrical conductivity (Figure 5A) and Seebeck coefficients (Figure 5B) enabled the determination of the power factor values (Figure 5C). The electrical conductivity values of the undoped SWCNT film slightly decreased as the temperature rose from 275 to 178 S/cm. The SWCNT film was composed of unsorted material, so it contained both semiconducting and metallic SWCNTs. The decrease in conductivity with the elevation in temperature indicates that the metallic character of the sample predominates over the semiconducting fraction of the material. This temperature dependence is associated with the charge-scattering effect in the material of metallic character, which becomes more noticeable with the increase in temperature. The same effect of a decrease in conductivity with temperature can be observed for all the dopants. It is important to note that once the temperature is decreased the high value of the electrical conductivity is restored. Therefore, the observed effect is unambiguously related to the increase of resistance of a metallic conductor at elevated temperature rather than dedoping.

Next, the Seebeck coefficients and Power Factors were measured for these materials to validate their utility for thermoelectrics. The Seebeck coefficient of the pure SWCNT material was 53 μV/K at room temperature and decreased to 47 μV/K at 100 °C. Most nitrogen dopants did not affect this value considerably because the Seebeck coefficient stayed within the 40–60 μV/K range regardless of the temperature. However, we noted three exceptions. Triazole, hexamethylenediamine (HMDA), and octylamine experienced a reduction in the Seebeck coefficients of SWCNTs to 22 μV/K, −36 μV/K, and −57 μV/K at room temperature, respectively. Thus, these doping species had the strongest interaction with the SWCNTs in terms of thermoelectric properties. In the case of the last two, even the sign of the Seebeck coefficient was changed, indicating the possibility of n-doping.

Finally, both at room temperature and above, all the dopants we studied caused the PF of the doped materials to be higher than that of the pristine SWCNT film. The most substantial increase in PF at room temperature was observed for imidazole and 2,4-dinitroaniline, reaching up to 275.5 μW/mK^2^ and 223 μW/mK^2^, respectively. As described earlier, with the temperature rise, changes in both electrical conductivity and the Seebeck coefficient were observed, which had a direct impact on the magnitude of PF values. Imidazole and 2,4-dinitroaniline turned out to be the best dopants at 100 °C with a PF of 294 μW/mK^2^ and 209 μW/mK^2^, respectively. These values are promising compared to state-of-the-art.^3^ Furthermore, the most significant difference in the value of PF between room temperature and 100 °C was observed for the SWCNT film doped with octylamine. Such a substantial decrease in the value of PF is connected to a notable reduction in the absolute value of the Seebeck coefficient above 70 °C. Three samples were selected for additional characterization to decipher the underlying reasons for the observed performance: imidazole (highest PF), octylamine (second highest PF, negative Seebeck coefficient), and hexamethylenediamine (negative Seebeck coefficient).

### 2.4 Microstructure

**Figure 6:**
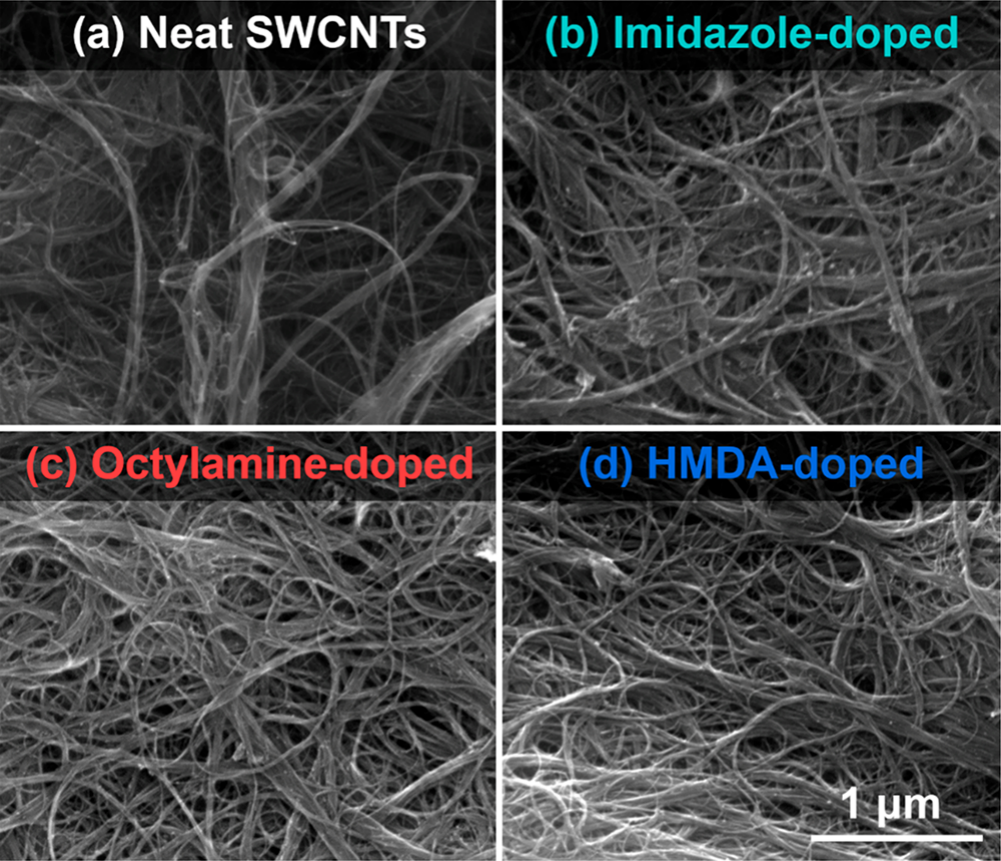
SEM micrographs of (a) neat SWCNT and that doped with (b) imidazole, (c) octylamine, and (d) hexamethylenediamine.

SEM was employed to study the microstructure of the material (Figure 6). The starting SWCNT material was composed of bundles of SWCNTs arranged in isotropic fashion due to the chosen manufacturing strategy. No signs of impurities in the form of carbonaceous deposits could be discerned, confirming the high quality of the SWCNTs. Voids were evident in the network, which promoted effective dopant infiltration.

Upon adding the dopants in organic solvents, a considerable fraction of voids were removed, or their size was reduced. This effect is the consequence of the elastocapillary-induced densification, which brings individual SWCNTs and their bundles closer to each other.^19,20^ Since these cavities are filled with air, one could postulate that the enhancement of electrical conductivity stemmed from removing these charge propagation obstacles. Nevertheless, all the nitrogen dopants were introduced in organic solvents. Some of them did not improve the electrical conductivity of the network, or the treatment reduced the conductivity. Hence, these possibilities can be excluded from consideration. We previously observed that the films prepared from the same raw SWCNTs using the reported manufacturing process experienced only minor improvement to the electrical properties or none upon densification.^17^ To fully understand the influence of the nitrogen-containing dopants on the thermoelectric properties of SWCNT films, we have performed DFTB and DFT-NEGF calculations on pristine and imidazole-, hexamethylenediamine-, and octylamine-doped SWCNTs.

### 2.5 Calculations

#### 2.5.1 Structural Properties and Stability

The influence of nitrogen compounds on different types of SWCNTs was studied in the DFTB approach using two types of SWCNTs: metallic and semiconducting SWCNTs of diameters comparable with the SWCNTs used in the experiments ((12,12), (20,0)) and also of smaller diameter SWCNTs ((5,5) and (10,0)) doped with imidazole (i), octylamine (o), and hexamethylenediamine (h), as shown in Figures S6 and S7. The last two nitrogen-containing compounds, aliphatic amines, were oriented along (A) and perpendicularly (B) to the symmetry axis of zigzag and armchair nanotubes to investigate whether the orientation of the alkyl chain depends on the orientation of the sp^2^ bonds in the SWCNT, as it does for halogens.^21^ The energy differences between these two configurations are very small (cf. *E*_ads/NC_ of hA(oA) and *E*_ads/NC_ of hB(oB) in Tables S1 and S3) but favor alignment of both amines along the symmetry axis of SWCNTs. In the case of small armchair ((5,5)) SWCNTs, the energetic difference between A and B configurations of octylamine is negligible at the DFTB level (Table S3), while DFT calculations clearly show that the A orientation is more favorable (*E*_ads/NC_ of oA is more negative than *E*_ads/NC_ of oB; see Table S4).

In contrast to the covalent functionalization of SWCNTs,^22−25^ the noncovalent nitrogen compound functionalization induces rather small changes in the structures of the SWCNTs. The increase in the coefficient of radius variation (CV) of SWCNTs, which measures their geometry changes with respect to undoped CNTs, is from one to two orders of magnitude smaller than those found for covalently functionalized SWCNTs. Among the nitrogen compounds considered, it is imidazole that causes the smallest changes in the structure of bigger SWCNTs. At the same time, the highest CV values are observed after doping these SWCNTs with octylamine (Figure S6). DFT calculations predict similar trends for smaller SWCNTs, while CV values calculated at the DFTB level are small and very similar for all these nanotubes (cf. CV values in Tables S3 and S4).

Although the interactions between octylamine and SWCNTs are the strongest among the nitrogen compounds considered (*E*_ads/NC_ is the most negative), the lowest-energy distance between imidazole and the nanotube is the smallest (Tables S1 and S3). The equilibrium optimal distance was observed between hexamethylenediamine in the A configuration and the SWCNT lateral surface among all investigated cases. Moreover, hexamethylenediamine oriented perpendicularly (hB) to the symmetry axis of metallic tubes is significantly closer to the SWCNT lateral surface than when aligned with these tubes (hA). An opposite behavior can be observed for octylamine. However, the differences between the oA-SWCNT and the oB-SWCNT distances are smaller than between hA-SWCNT and hB-SWCNT. The space between all considered nitrogen compounds and the small metallic SWCNT is smaller than between them and the bigger metallic SWCNT (Tables S1 and S3). Note that the nitrogen compound–SWCNT distances calculated at the DFTB level are larger than those obtained at the DFT level (Tables S3 and S4).

Since the experimental samples are predominantly composed of metallic nanotubes, the impact of nitrogen compound concentration was computed for the (12,12) nanotube. Two more nitrogen compounds were added to the (12,12) + hB and (12,12) + i systems, creating (12,12) + hB_3_ and (12,12) + i_3_ systems, respectively (see top panels in Figures S8 and S10). The two additional molecules were placed close to the previously physisorbed nitrogen compounds. After full geometry optimization, all three hexamethylenediamines are found between A and B configurations, creating an obtuse-angled triangle above the lateral surface of the nanotube with N atoms of neighboring amines facing each other. Imidazoles create a chain, in which the N atoms are closer to each other than to the C atoms from another azole. To check whether the final orientation of hexamethylenediamines depends on their concentration, a (12,12) + hB_7_ system with six additional amines was prepared. After full optimization, seven hexamethylenediamines remained oriented perpendicular to the tube axis, creating a ring around the nanotube. It transpires (Table S1) that physisorption of more nitrogen compounds to the lateral surface of the (12,12) nanotube is energetically preferred (*E*_ads/NC_ becomes more negative), but only the energetic stability of SWCNTs doped with the hexamethylenediamine increases with increasing concentration of molecules (*E*_bind/N_ of (12,12) + hB_3_ and (12,12) + hB_7_ are more negative than that of (12,12) + hB, while *E*_bind/N_ of (12,12) + i_3_ is less negative than that of (12,12) + i). Furthermore, the distance between the nanotube lateral surface and the nitrogen compounds decreases when more molecules are physisorbed to the SWCNT. Surprisingly, the tube deformation (CV value) after absorption of three imidazoles and seven hexamethylenediamines becomes smaller than it is after physisorption with only one molecule.

#### 2.5.2 Electronic Properties

**Figure 7:**
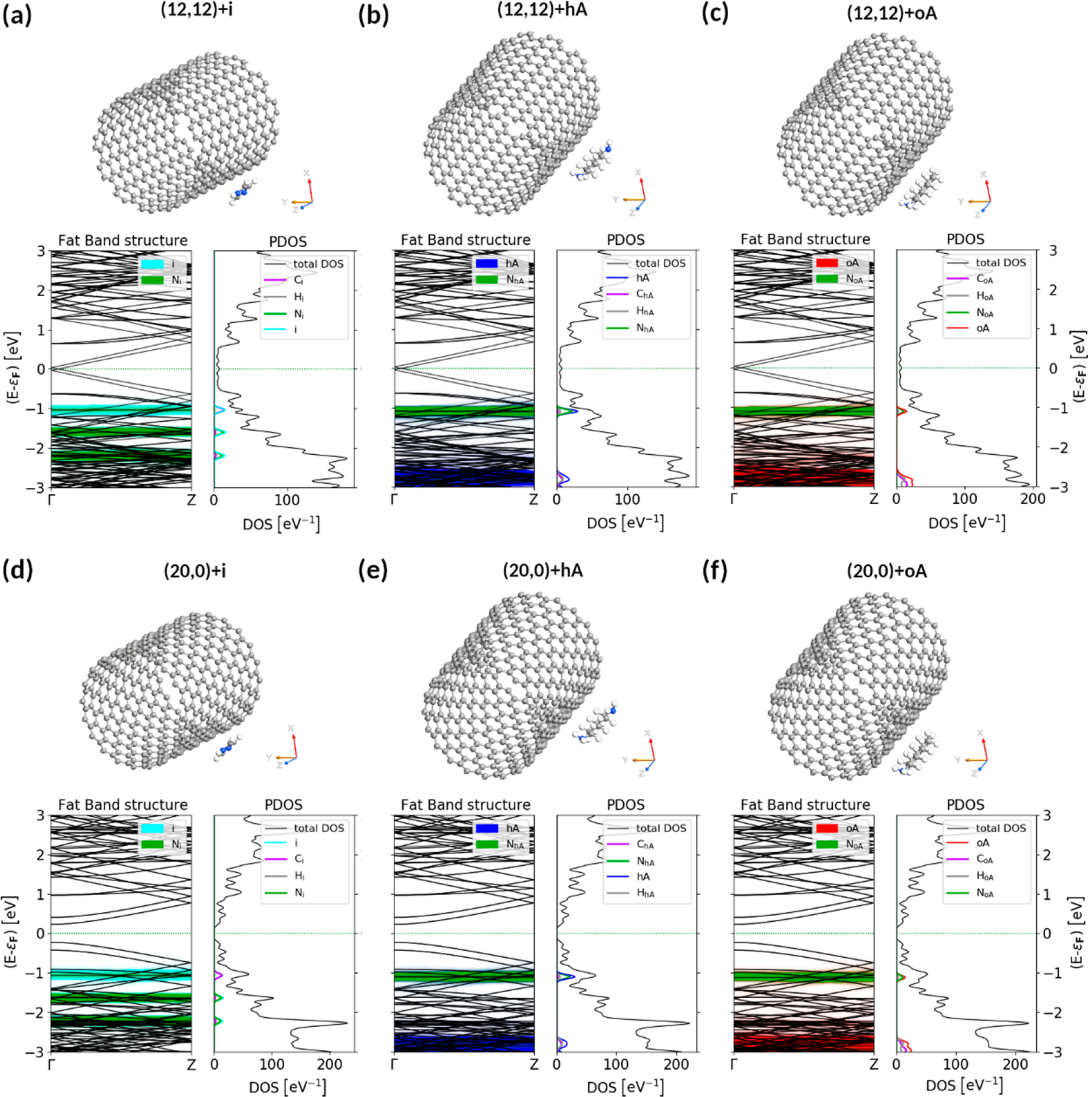
DFTB-computed electronic properties of the fully optimized most stable configurations of (12,12) and (20,0) SWCNTs doped with (a,d) imidazole, (b,e) hexamethylenediamine, and (c,f) octylamine. Octylamine (oA) and hexamethylenediamine (hA) are oriented along the SWCNT symmetry axis. The fat band structures along Γ → Z of the Brillouin zone together with the projected density of states on dopant N, dopant C, and dopant H species (PDOS). Atomistic cross-sectional views of the fully geometry-optimized systems are shown above. N and C atoms are depicted in blue and gray, while H atoms are shown in white. The Fermi level was shifted to 0.

To fully understand the character and origin of the interactions between the SWCNT and nitrogen compounds, it is necessary to analyze the electronic properties of the doped systems. Similarly to the covalent functionalization of nanocarbons with amines,^23,24,26,27^ noncovalent nitrogen compound doping of SWCNTs introduces additional impurity bands (Figures 7, S11A,B,D,E, and S12A,B,D,E), induces charge transfer (Figures S11C,F and S12C,F), and reduces the energy band gap of the small semiconducting SWCNT (Tables S3 and S4).

However, there are some differences. The impurity bands resulting from nitrogen compounds physisorbed to the lateral surface of nanotubes lie further from the Fermi level (below −1 eV). They are also flatter than those produced on chemisorption. The reduction in the energy band gap of small semiconducting nanotubes is also much smaller (Table S3) when nitrogen compounds interact only weakly with the SWCNT backbones. For bigger semiconducting SWCNTs, one can observe an increase in the energy band gap after imidazole and hexamethylenediamine physisorption compared to the pristine (20,0) SWCNT (Table S1).

In the case of octylamine, the final effect depends on its orientation around the nanotube. In the B configuration, octylamine behaves in the same way, but when aligned with the nanotube (oA), it slightly reduces the energy band gap of the pristine system. It is also worth noting that DFT calculations (Table S4) show a small opening of the energy band gap in (5,5) SWCNTs after doping with all considered nitrogen compounds.

A closer inspection of the electronic properties of systems doped with all nitrogen compounds also reveals differences between these molecules. Upon doping with imidazole (Figure 7A,D), three flat bands appear in the range of [0, −2] eV below the Fermi level, from which the two deeper bands mostly originate from N atoms, while the shallower band, around −1 eV, comes primarily from imidazole C atoms and only to a smaller extent from imidazole N atoms. At a similar energy from the Fermi level (−1 eV) lies the first impurity band introduced by each amine (Figure 7B,C,E,F), but it is nitrogen, which contributes the most to these bands. The rest of the amine impurity bands lie below the two imidazole impurity bands (below −2.2 eV) and are broader than them. The density of states (DOS) peak corresponding to the first hexamethylenediamine (octylamine) impurity band is higher (lower) than that below −2.5 eV, while the number of imidazole-induced states is evenly distributed over three visible levels.

Moreover, the differences in the interactions between aromatic azoles and SWCNTs and between aliphatic amines and SWCNTs are also clearly visible in the electron difference density (EDD) maps (Figures S11 and S12). These maps show the difference between the self-consistent valence charge density and the superposition of atomic valence densities. The blue region indicating a deficiency of electrons is visible all along the aliphatic amine chain and SWCNTs, while the red region showing an excess of electrons is localized only between the amine nitrogen and SWCNT. Due to the azole geometry, the red and blue regions associated with sp^3^ and sp^2^ nitrogen atoms are adjacent on the ring, creating a zwitterion structure that is highly efficient for extracting electrons from the nanotube. As the macroscopically averaged EDD plots along the symmetry axis of nanotubes show, changes in the SWCNT electron density induced by doping with imidazole are much more pronounced (cf. panels c and f in Figures S11 and S12) than by doping not only with octylamine that has one amine group but also with hexamethylenediamine, which contains two amine groups. Note that DFT calculations show smaller differences between the three types of dopants, especially for semiconducting tubes.

**Figure 8:**
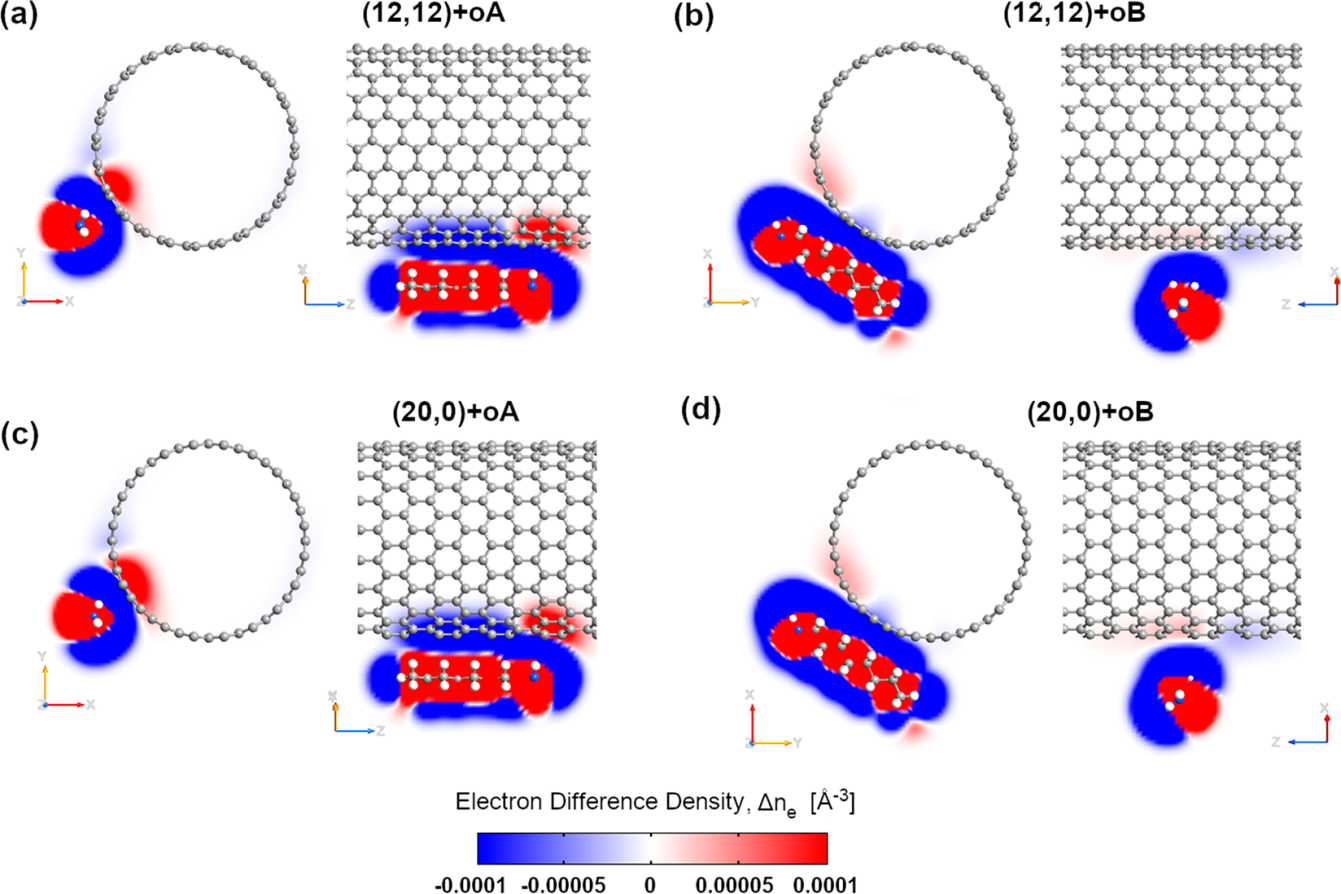
Electron difference density (EDD) maps of fully optimized (a,b) (12,12) and (c,d) (20,0) SWCNTs doped with octylamine oriented (a,c) along and (b,d) perpendicular to the CNT symmetry axis. The electron difference density maps show the difference between the self-consistent valence charge density and a superposition of atomic valence densities. The cut planes are taken through the N atom. N and C atoms are depicted in blue and gray, while H atoms are shown in white. The blue regions indicate a deficiency of electrons, while red regions show an excess of electrons.

The most interesting effect is visible when one compares the changes induced by the same type of amine but differently oriented around the tube. The comparison between macroscopically averaged 1D projections of EDD on the *z*-axis of (12,12) and (20,0) SWCNTs doped with octylamine (Figure S8) shows a stark difference between the A and B configurations. The macroscopically averaged EDD of SWCNT + oB looks very similar to the macroscopically averaged EDD of SWCNT + i. The differences in the interaction between both octylamine configurations and the SWCNTs are also clearly visible in the EDD maps (see Figures 8 and S9), which display the difference between the self-consistent valence charge density and the superposition of atomic valence densities. EDD maps, in agreement with Bader charge population analysis, show that hole transfer induced by oA to the nanotube is much higher than induced by oB. The blue regions indicate that the deficiency of electrons around the nanotube carbon atoms is much bigger when octylamine is aligned with the nanotube (oA) than when it is perpendicular to the SWCNT axis (oB). In the latter cases (oB), the ratio of the red regions (excess of electrons) to the blue regions around the nanotube carbon atoms completely changes. This result suggests that the charge transfer from octylamine to SWCNT depends on its orientation around the lateral surface of the nanotube. Hence, the doping level of the nanotube can be tuned by varying the orientation of octylamine.

Analysis of the electronic properties of (12,12) nanotubes doped with different concentrations of hexamethylenediamine (Figure S10) and imidazole (Figure S13) showed that the number of doping-induced bands is proportional to the concentration of nitrogen compounds. Thus, increasing the concentration of hexamethylenediamine simply increases the height of the DOS peaks, resulting from amine at about −1 eV and below −2.5 eV from the Fermi level. On the other hand, the physisorption of more imidazole molecules inserts additional states in between the previously introduced states, broadening the energy range in which they appear.

Applying strain to SWCNTs can cause a charge reorganization and also produces a change of the metallic/semiconducting character of pure nanotubes.^28−30^ This effect can also be produced by covalent functionalization of nanocarbons, as dopants make global changes to SWCNTs and graphene structures.^24,31^ Our theoretical investigation concerns the properties of isolated nanotubes in vacuum, so it is important to check how changing the distance between nitrogen compounds and SWCNTs affects the electronic properties of these systems. To do so, we analyzed the electronic properties of (12,12) nanotubes doped with imidazole and octylamine that were placed closer (*d*_1_) and further (*d*_2_) from the SWCNT lateral surface than they were after complete geometry optimization (Figure S14, top panel). After moving dopant molecules away or toward SWCNTs, the systems were again optimized, but the position of the N atom in the molecule (one of two in case of the imidazole) and *z*-coordinates of the most distant two neighboring rows of SWCNT carbon atoms were kept fixed within the supercell. Changing the distance between both dopants and the SWCNT slightly opened the energy band gap of (12,12) SWCNTs. It also induced some changes in the charge distribution around the tubes (Figure S15), but these changes are minimal compared to the differences caused by doping with different agents.

#### 2.5.3 Thermoelectric Properties

**Figure 9:**
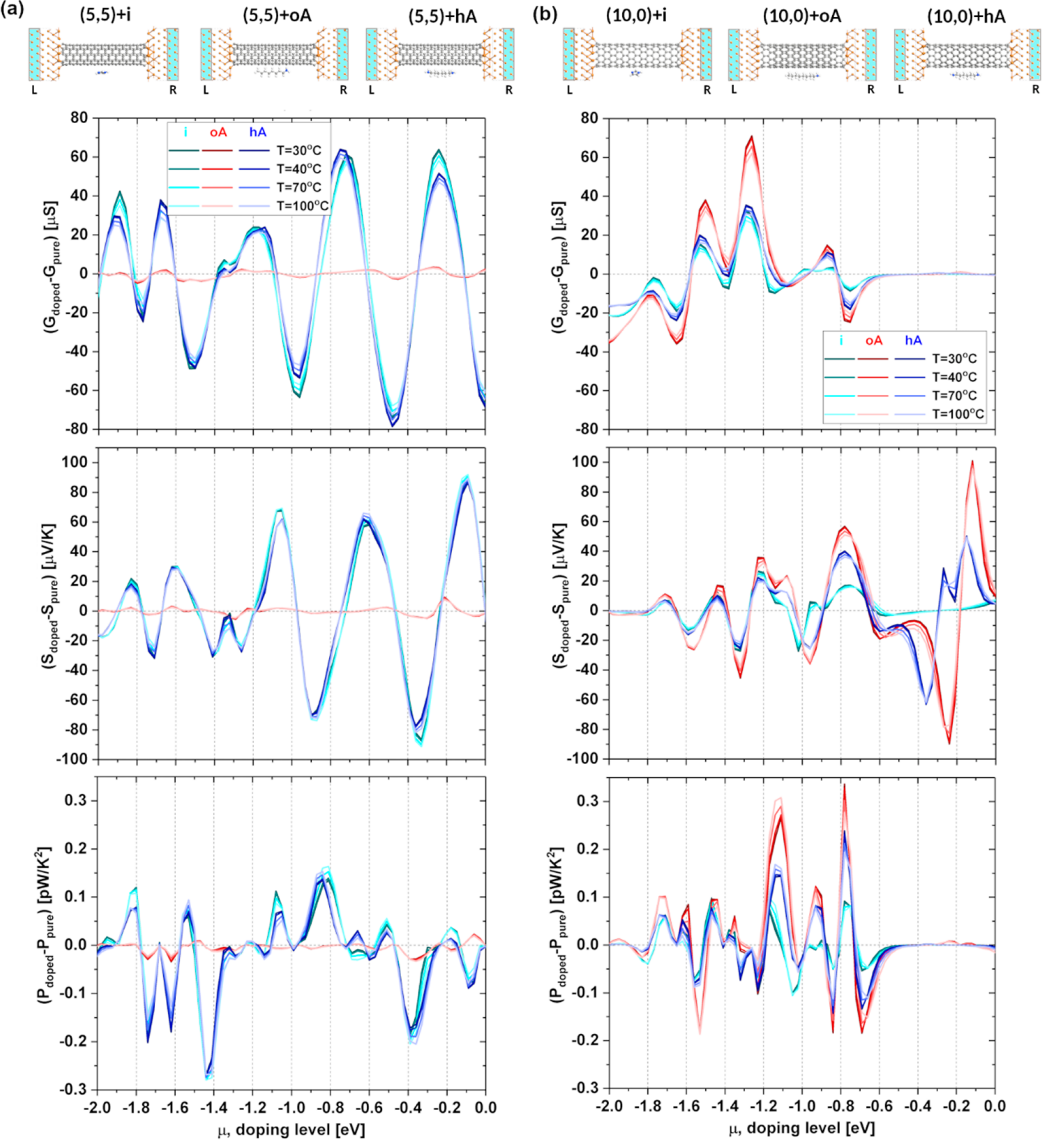
DFT-computed changes in thermoelectric properties of (a) metallic (5,5) and (b) semiconducting (10,0) SWCNTs positioned between metallic electrodes after doping with imidazole, octylamine, and hexamethylenediamine. Conductance (*G*), Seebeck coefficient (*S*), and power factor per SWCNT (*P*) changes with respect to the systems containing pristine nanotubes are plotted as a function of doping level (μ) for four different temperatures: 30, 40, 70, and 100 °C. The atomistic side views of the models used for the transport calculations are presented at the top of the figure. N, C, and H atoms are depicted in blue, gray, and white, while Cu atoms are shown in orange. The semi-infinite electrodes consisting of perfect copper are highlighted in blue.

To further understand the physical mechanisms governing the thermoelectric performance of the doped SWCNT films shown in Figure 5, we performed transport calculations for a selection of the systems previously analyzed. DFT-NEGF calculations were done on the device models shown in Figure 9 (top panel), which are the smallest possible systems that allow us to qualitatively reproduce the experimentally observed trends in the thermoelectric properties of pristine SWCNTs.^17^

The pristine and doped (5,5) and (10,0) SWCNTs were coupled to Cu electrodes. Copper as well as silver, used in experiments, interact rather weakly with SWCNTs (see Figure S16A). Hence, the intrinsic electronic structure properties of SWCNTs are preserved to a large extent when contact is made.^32,33^ Charge transfer between Cu and the nanotube or between Ag and the nanotube produces band bending, which enables the valence band edge of the SWCNT to align with the Fermi level of the electrode.^34^ Unfortunately, creating an interface between these metals and the nanotube disturbs the SWCNT structure. The reduced sp^2^ hybridization of the SWCNT causes a decrease in the conductance of metallic SWCNTs with respect to the conductance of SWCNTs coupled to the SWCNT electrodes.^17,32^ On the other hand, Cu and Ag contacts introduce additional states in the energy gap region of semiconducting SWCNTs, the so-called metal-induced gap states,^35,36^ inducing metallization of these nanotubes.^34^ Note that qualitative changes to the thermoelectric properties of SWCNTs induced by Cu and Ag electrodes are similar (see Figure S16B).

Figure 8A,B shows the doping-induced changes in the electrical conductance, in the Seebeck coefficient, and in the power factor per SWCNT as a function of chemical potential for the systems containing metallic and semiconducting SWCNTs, respectively. Due to the difficulty of defining the cross-sectional area of SWCNTs, we decided to calculate only the electrical conductance (*G*) instead of conductivity (γ) to obtain the power factor per nanotube (*P*)^37,38^ rather than the absolute power factor (PF) presented in Figure 5. Since the pretreatment makes SWCNT systems p-doped,^17,37^ only negative chemical potential values (the p-doping region) are displayed. Since the chemical potential of CNT film samples can be tuned by changing the pretreatment conditions such as used solvents,^39^ all thermoelectric properties are presented as a function of chemical potential. The doping of metallic and semiconducting SWCNTs with imidazole, octylamine, and hexamethylenediamine leads to clearly visible differences for each thermoelectric property. However, it should be noted that the final effect of the doping with all agents depends on the initial treatment of the system (its chemical potential). It is possible to obtain either a reduction or increase in G, S, and P of all considered systems for a given temperature, depending on the chemical potential.

A detailed analysis of Figure 9 shows that the smallest impact on the *G*, *S*, and *P* of the metallic tube has octylamine oriented along the CNT symmetry axis (oA). Changes in the thermoelectric properties of (5,5) SWCNTs induced by doping with hA and i are similar, and their absolute values are much bigger than those caused by oA. The opposite behavior can be observed for semiconducting (10,0) SWCNTs. Octylamine aligned with the nanotube symmetry axis induces the biggest changes in *G* of (10,0) SWCNTs, while imidazole and hexamethylenediamine (hA) have the smallest impact on it, but only for μ ∈ (−2, −0.6) eV. Similar observations can be made for the doping-induced changes in *S* and *P*. The only difference is seen in the Seebeck coefficient of the semiconducting SWCNT. The differences in the Seebeck coefficient of (10,0) SWCNTs induced by both amines (oA and hA) with respect to the undoped system are more pronounced for μ ∈ (−0.6,0) eV than for μ ∈ (−2, −0.6) eV.

Interestingly, the magnitude of the changes in the thermoelectric properties of nanotubes doped with differently oriented octylamine (A and B configurations) is comparable with the magnitude of the changes induced by doping SWCNTs with different agents (cf. Figures 8 and S17). These results indicate that alignment of octylamine in the SWCNT networks, even when the compounds are only physisorbed to SWCNTs, controls the resultant doping level and, thus, the thermoelectric properties of the system. To verify this effect on bigger SWCNTs, we performed DFTB-NEGF calculations on doped (12,12) SWCNTs coupled to pristine (12,12) SWCNT electrodes. A comparison between (12,12) SWCNTs doped with octylamine oriented along (oA) and perpendicular (oB) to the symmetry axis of the SWCNTs (Figure S18B) suggests that the impact of octylamine orientation on *G*, *S*, and *P* of (12,12) SWCNTs is restricted to a rather narrow range of chemical potential. More significant differences between configurations A and B in the broader range of μ are visible for hexamethylenediamine (Figure S18A). Increasing the concentration of nitrogen compounds should have a more pronounced effect on the thermoelectric properties of larger-diameter metallic nanotubes than changing the orientation of amines (cf. magnitude of *G*, *S*, and *P* changes in Figures S18 and S19). The DFTB-NEGF calculations also show that changing the distance between the doping agents and the lateral surface of SWCNTs may affect their thermoelectric properties (Figure S14). However, changes in *G*, *S*, and *P* in the (12,12) + oA system are only significant in a rather narrow range of chemical potential. Smaller changes of thermoelectric properties of (12,12) SWCNTs, but over the broader range, can be observed in the case of imidazole when its distance from the nanotube was varied.

**Figure 10:**
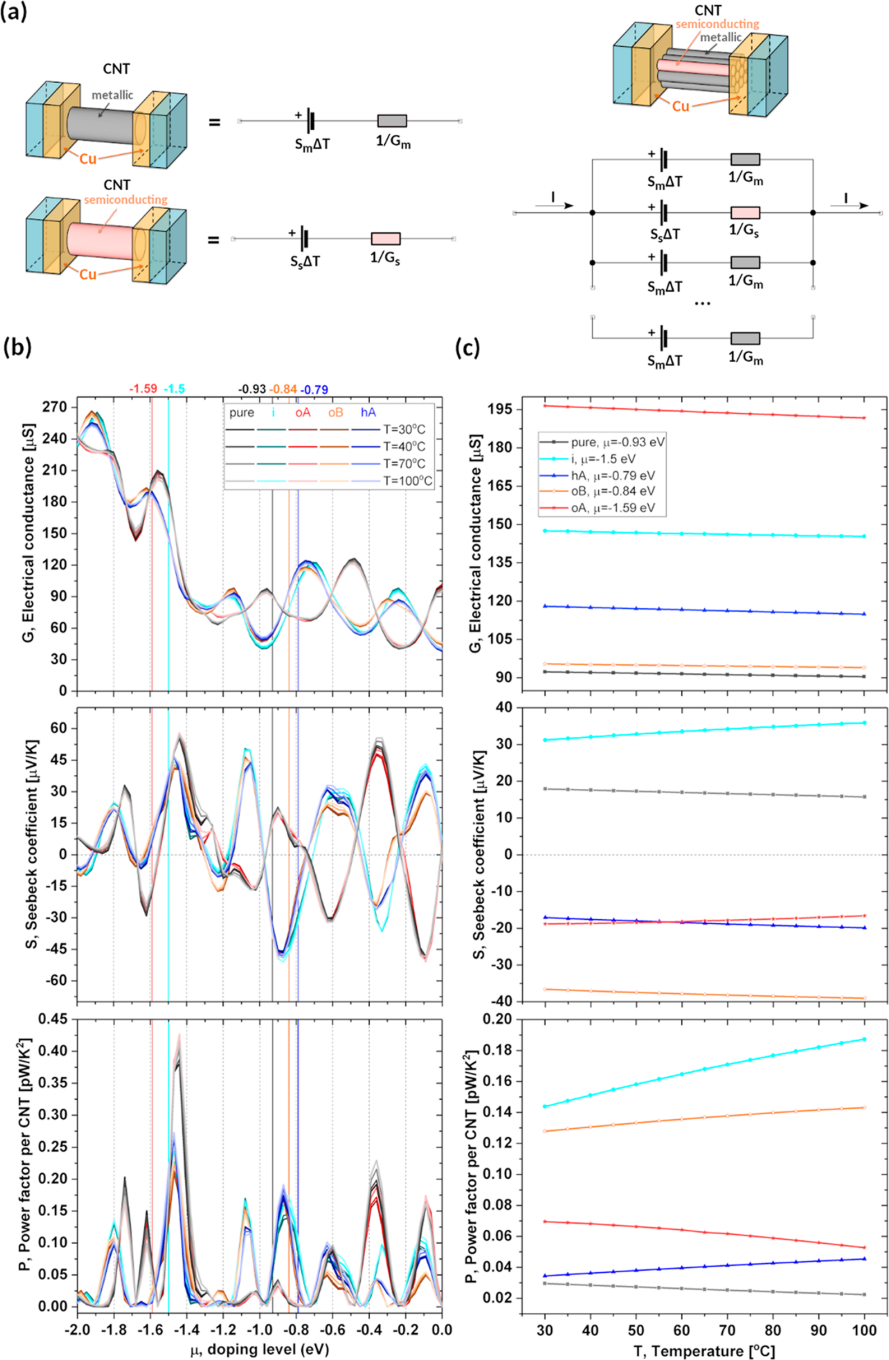
DFT-computed thermoelectric properties of pristine and doped mixed parallel SWCNT circuits placed between metallic electrodes. (a) The 3D visualization of the SWCNT bundle model made of 90% metallic (5,5) and 10% semiconducting (10,0) SWCNTs based on the model published by Hayashi et al.^38^ The equivalent circuits for both types of SWCNTs and the parallel connection model are shown next to and below the 3D visualizations. (b) Conductance (*G*), Seebeck coefficient (*S*), and power factor per SWCNT (*P*) are plotted as a function of doping level (μ) for four different temperatures: 30, 40, 70, and 100 °C. (c) *G*, *S*, *P* at different temperatures for pristine and imidazole (i), octylamine (o), and hexamethylenediamine (h) doped SWCNT films. Both systems containing octylamine, oriented along (oA) and perpendicular (oB) to the SWCNT symmetry axis, are shown.

Having described the impact of different nitrogen compounds on the thermoelectric properties of isolated SWCNTs, we can now focus on their impact on the thermoelectric properties of the SWCNT films. We used a simple model of mixed parallel SWCNT circuits (Figure 10A) that allows us to qualitatively reproduce the thermoelectric properties of mostly metallic pristine SWCNT films.^17^ As in the experimental samples, the SWCNT film models contain 90% of metallic SWCNTs. Figure 10B shows the computed thermoelectric properties as functions of chemical potential for SWCNT films made of pristine/doped (5,5) and (10,0) SWCNTs for four different temperatures. Figure 10C shows the thermoelectric properties of these systems at different temperatures for values of the chemical potential that reproduce experimental trends presented in Figure 5. For a chemical potential of −0.93 eV (see black vertical lines in Figure 10B), we obtain a decrease in all thermoelectric properties of undoped SWCNT films with increasing temperature (see gray lines in Figure 10C). The chemical potential values were obtained from the fitting of *G*(*T*), *S*(*T*), and *P*(*T*) functions to the experimental γ(*T*), *S*(*T*), and PF(*T*).

For imidazole- and hexamethylenediamine-doped systems, we also identified μ values, −1.5 eV (light blue vertical lines in Figure 10B) and −0.79 eV (dark blue vertical lines in Figure 10B), respectively, which give the same qualitative behavior as our experiments (Figures 5 and 10C). Our calculations clearly show that imidazole is a p-type dopant, while hexamethylenediamine is an n-type dopant. In the case of octylamine, none of the considered models, oA nor oB, fully reproduce the peculiar nonmonotonic behavior of *S*(*T*) and PF(*T*) observed in our experiments. As can be seen in Figure 10C, *G* and *P* of a SWCNT film doped with oA decrease with increasing temperature, while *S* of that system increases with temperature. This resembles the high-temperature (above 70 °C) behavior of experimental samples doped with octylamine. Moreover, the Seebeck coefficient of the oA model becomes more negative than that of the hA model but at a lower temperature than that observed in experiments (ca. 60 °C vs ca. 95 °C). On the other hand, *G* and *S* of the SWCNT film model doped with oB decrease with temperature. At the same time, the *P* value of that system increases with temperature, reproducing the low-temperature (30–70 °C) experimental trends. The discrepancies from the experiments can be seen in *G* of both models (Figures 10C (top) and 5A). The electrical conductance of the oA model is higher than of the i model, while in experiments, imidazole-doped samples are characterized by the highest electrical conductivity. The electrical conductance of the oB model is smaller than that of the h model, opposite from that observed in experiments. Relatively small differences between modeling and experiments can be explained by the simplicity of the models considered (only two types of small-diameter tubes organized in parallel circuits) as well as the fact that inelastic electron scattering, electron localization effects,^37^ and SWCNT–SWCNT junctions^40^ are neglected.

The modeling results suggest that the orientation of octylamine around the SWCNT changes with temperature. To confirm this hypothesis, we performed short DFTB-MD simulations. The NVT (Nosé–Hoover) ensemble calculations of SWCNTs doped with octylamine oriented along and perpendicularly to the symmetry axis of SWCNTs showed that a change of amine alignment during annealing is possible. Electronic transport simulations also predict that different octylamine orientations on the SWCNT surface are associated with different doping levels. As indicated in Figure 10B by red and orange vertical lines, the perpendicular orientation of octylamine (oB) produces an n-type doped system with respect to the pristine SWCNT film, while alignment of octylamine with the SWCNT axis (oA) shifts the doping level to more negative values (inducing p-doping). Since the orientation of octylamine can be controlled by temperature, the doping level and character can also be tuned this way. These results fully explain the experimental trends and underpin the argument that the orientation of octylamine is one of the key factors dictating the electronic behavior of the network.

## 3 Conclusions

While analyzing the structure of various amines and cyclic compounds containing a nitrogen atom, we observed that the dopant structure has a significant impact on the ability to enhance the electrical conductivity of SWCNT films. In the case of aliphatic amines, it was found that the length of the aliphatic chain and the degree of steric hindrance in the dopant influence the SWCNT doping capabilities. For octylamine, the strongest interacting dopant whose doping character depends on temperature, the thermoelectric properties of SWCNT films can be controlled by changing the alignment of the octylamine around the SWCNTs.

Furthermore, in the case of nitrogen-containing heterocyclic compounds, we observed that the electrical conductivity of SWCNT films doped with these compounds correlates with the Hammett substituent constants and the p*K*_a_ value. While an increase in p*K*_a_ for pyrazine and azoles was favorable for enhancing the electrical conductivity of SWCNTs, the opposite was true for anilines and pyridines. The selected dopants had a significant impact on the electrical and thermoelectric properties of the materials. We observed a significant increase in the value of electrical conductivity for all of them, which was increased by a factor of 3 in the best-case scenario (imidazole). Moreover, certain doping species changed the sign of the Seebeck coefficient, which indicates a strong influence on the charge transport properties in the material. The doping level and its type (n-type or p-type) strongly depend on the changes in SWCNT charge density induced by a specific nitrogen compound and on the number of electronic states introduced that can contribute to the transport. Lastly, the highest PF value of 275 μW/mK^2^ at room temperature and 293 μW/mK^2^ at 100 °C was recorded for SWCNTs doped with imidazole.

Our results demonstrate that the rich nature of organic nitrogen compounds can be exploited to tune the properties of nanocarbon systems. Since these chemical compounds can be made on-demand in numerous configurations, they can be designed and applied to these materials to obtain the necessary product parameters for selected applications. From the fundamental research point of view, the findings presented give strong proof that the Fermi level in these materials can be easily modulated when a nitrogen dopant of appropriate p*K*_a_, orientation with respect to the SWCNT, and concentration are incorporated.

## 4 Experimental Section

### 4.1 Materials

Thin free-standing films were made from high-quality SWCNTs of about 1.6 ± 0.4 nm in diameter (Tuball, OCSiAl). For the manufacture of films, we used toluene and acetone as solvents (pure p.a. class, ChemPur, Poland) and ethyl cellulose as an interim binding agent (EC, pure p.a. class, ethoxyl content 48%, Acros Organics, Poland).

A range of nitrogen doping agents were explored: propylamine, butylamine, octylamine, isopropylamine, *tert*-butylamine, dibutylamine, dicyclohexylamine, triethylamine, tributylamine, *N*-methylbenzylamine, diphenylamine, *N*,*N*-dimethyl-1-benzylamine, allylamine, ethanolamine, aminopropan-3-ol, aminopropan-2-ol, tris(hydroxymethyl)aminomethane, diethanoloamine, ethylenediamine, hexamethylenediamine, hexamethylenetetraamine, aniline, *p*-phenylenediamine, 3-nitroaniline, 2,4-dinitroaniline, 2-methylaniline, 4-aminobenzoic acid, acetanilide, pyridine, 4-hydroxypyridine, 3-cyanopyridine, 2-acetylpyridine, 2-amino-3-methylpyridine, imidazole, benzimidazole, triazole, benzotriazole, tetrazole, and pyrazine. They were purchased from Acros Organics, Alfa Aesar, Sigma-Aldrich, or Avantor. All of them were of pure p.a. class.

### 4.2 SWCNT Film Preparation

To measure the thermoelectric properties of SWCNTs doped with nitrogen compounds, we manufactured their macroscopic ensembles. We used the method of making SWCNT films described previously.^41^ Briefly, the process involved to prepare a SWCNT dispersion within an ice-cold mixture of toluene and acetone (1:1) was facilitated by an EC binder. Then, the dispersion was deposited onto Kapton foil, forming an SWCNT film upon evaporation of the solvent. Subsequently, the film was peeled off the substrate, and the EC was removed by high-temperature annealing. Thin self-standing SWCNT films free of polymer were produced as a result and used in the study.

### 4.3 Doping of SWCNT Films

We doped the films by dipping the material in solutions containing the nitrogen doping agents. SWCNT film strips of ca. 3 × 55 mm were immersed in 0.1 M solution of the dopant in acetone for 30 s. After immersion, the SWCNT films were dried in a vacuum desiccator. The whole process of manufacturing the SWCNT films and nitrogen doping is illustrated schematically in the Supporting Information (Figure S21).

### 4.4 Characterization

Electronic and structural modifications in the films were studied by Raman spectroscopy. Spectra were collected (Renishaw, λ = 514 nm laser, integration time of 10 s) from 0 to 3500 cm^–1^. The measurement was conducted each time in several specimen areas for an extended integration time to guarantee the statistical significance of the generated data and appropriate signal-to-noise ratio.

The changes in electrical conductivity caused by doping were gauged using the 4-point method with a source meter (Keithley 2450). A 100 mA electric current was employed to avoid heating the specimens. The electrical conductivity for all the doped films was measured at room temperature. Measurements were also done at elevated temperatures (40 °C, 70 °C, and 100 °C) in the case of the most promising nitrogen-doping compounds. In these cases, the samples were heated on a hot plate, while the temperature was verified with an infrared thermometer (FLIR ETS 320).

The Seebeck coefficients were determined using a custom-made setup (SeebCam, LBR, Lublin, Poland) in the temperature range from 30 to 100 °C. SWCNT film samples (2 × 50 mm) were placed on a board, which was situated in a sealed chamber to eliminate the effect of convection. Both ends of the sample were interfaced with temperature sensors and resistive heaters. Conductive silver paint was used to ensure electrical contact between the sample and the setup. Then, the electric potential difference between the sample ends was measured using a temperature gradient of 5 °C in the temperature range specified above. Five measurements were carried out for each sample. The obtained results were averaged, and the uncertainty was calculated. The PF values were calculated based on the registered values of electrical conductivity and Seebeck coefficients.

The thermal stability of doped SWCNT films was studied by using a thermogravimetric analyzer (Mettler Toledo TGA/DSC 1 STAR) in the temperature range from 25 to 1000 °C. The samples were characterized in the flow of air (30 mL/min) with the heating rate of 10 °C/min.

The microstructure of the selected specimens was inspected by scanning electron microscopy (SEM, FEI Quanta 250 FEG, 15 kV acceleration voltage) under high vacuum conditions.

The p*K*_a_ values for all the compounds were collected from the literature,^42−46^ and the Hammett constant (σ) for the group of compounds classified as aniline and pyridine derivatives was calculated using the following formulas.

For aniline derivatives:^47,48^
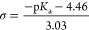


For pyridine derivatives:^49−51^
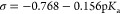


### 4.5 Modeling

The structural and electronic properties of pure and doped SWCNTs were calculated using the density functional based tight-binding (DFTB) method^52^ with Slater–Koster parametrization for C, N, and H atoms (auorg-1–1)^53,54^ as implemented in QuantumATK.^55−57^ The DFTB method is an efficient method that provides a useful fundamental understanding of large systems, such as nanometer-sized SWCNTs, at the atomic level.^21,58^ A self-consistent charge correction that takes into account charge fluctuations due to interatomic electron–electron interactions was included in the calculations. The Brillouin zone was sampled using (3 × 3 × 7) k-points, while the density mesh cutoff for real-space integrals was set to 30 Ha. For the band structure and density of states calculations, the sampling was increased to (7 × 7 × 11) k-points and 50 Ha. The self-consistent field (SCF) cycle was iterated for all calculations until the density matrix elements changed by less than 10^–7^ per iteration. The models presented in Figures S6 and S7 were optimized until the maximum force converged to lower than 0.005 eV/Å, and the maximum stress changed by less than 0.1 GPa per iteration. The computed structural and electronic properties for SWCNTs of about 1.6 nm in diameter ((20,0) and (12,12)) are presented in Table S1, whereas Table S3 contains computed parameters for SWCNTs of diameter below 1 nm ((10,0) and (5,5)).

Coherent electron transport calculations for (5,5) and (10,0) SWCNTs were performed employing density functional theory (DFT) in combination with the nonequilibrium Green’s function method (NEGF) as implemented in QuantumATK.^55,59^ Calculations were carried out in the generalized gradient approximation (GGA) of DFT using standard Perdew–Burke–Ernzerhof (PBE) parametrization^60^ and single-ζ plus polarization numerical basis (SZP) sets of orbitals localized on atoms. London dispersion interactions between the nitrogen compounds and the SWCNTs were included in the total binding energy using the methodology proposed by Grimme.^61^ Pure and doped SWCNTs comprising the central scattering regions of device configurations were coupled to two semi-infinite metallic electrodes, as shown in Figure 9 (top panel). The interfaces between the Cu(100) surfaces and the SWCNT open ends were fully relaxed until a maximum force converged to lower than 0.01 eV/Å, and the maximum stress changed by less than 0.1 GPa. The Brillouin zone of the two-probe system was sampled using a 2 × 2 × 101 Monkhorst–Pack scheme. The transmission spectra were calculated using increased 7 × 7 k-point sampling in the [−3, 3] eV range within 1201 points.

The electrical conductance and the Seebeck coefficient were calculated using linear response theory as^56,62^
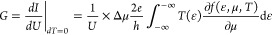
and
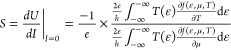
where *I* is the electrical current through the device under a finite bias voltage, U; *T*(ε) is the energy-resolved transmission function; *f*(ε,μ,*T*) is the Fermi–Dirac electron distribution; μ = ε_F_ ± *eU*/2 represents the electrochemical potential of the electrode; ε_F_ is the Fermi energy; and *T* is the temperature of the electrode. In the ballistic regime, the electrical current can be calculated using the Landauer–Büttiker formula:^63^, where indexes L and R refer to left and right electrodes; *h* is the Planck constant; *e* is the electron charge; and *k*_B_ is the Boltzmann constant. The transmission function for electrons with energy ε incident in the central region of the device can be expressed by the retarded Green’s function , where  is the broadening function for the left (right) electrode, and  is the corresponding self-energy calculated in the iterative self-consistent approach. The power factor per SWCNT is defined as GS^2^.^37,38^

Thermoelectric properties of SWCNT films were calculated using a simple model of mixed parallel SWCNT circuits.^37^ The conductance and the Seebeck coefficient in the parallel model containing 90% metallic SWCNTs are defined as
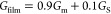
and
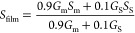
where *G*_m(s)_ and *S*_m(s)_ are the conductances and Seebeck coefficients of metallic and semiconducting SWCNTs, as shown in Figure 9A.

## Supporting Information Available

The method of doping of SWCNTs with nitrogen compounds, a list of used dopants, techniques used for modeling, Raman spectra and thermograms of selected doped SWCNT films, atomistic cross-sectional and side views of fully optimized SWCNTs, DFTB-computed structural parameters, adsorption energies per nanotube carbon atom, binding energies per atom, energy band gaps, Fermi levels, the valence band maximum (VBM) and conduction band minimum (CBM) with respect to the Fermi levels of fully optimized pristine and nitrogen compound doped SWCNTs, DFT- and DFTB-computed electronic and thermoelectric properties, and preparation of nitrogen compound doped SWCNT material (PDF)
