# Supplementary material for: Doping Engineering of Single-Walled Carbon Nanotubes by Nitrogen Compounds Using Basicity and Alignment
Source: ACS Appl Mater Interfaces. 2022 May 18;14(22):25861–77. doi: 10.1021/acsami.2c00970 (PMC9185683; doi:10.1021/acsami.2c00970)
Supplement: Supplementary file 1 — am2c00970_si_001.pdf [file am2c00970_si_001.pdf]

Supporting Information

Doping Engineering of Single-Walled Carbon Nanotubes by Nitrogen Compounds Using Basicity and Alignment

Bogumiła Kumanek[a,*,‡], Karolina Z. Milowska[b,c,d*,‡], Łukasz Przypis[a], Grzegorz Stando[a], Karolina Matuszek[e], Douglas MacFarlane[e], Mike C. Payne[b], Dawid Janas[a,*]

[a] Department of Organic Chemistry, Bioorganic Chemistry and Biotechnology, Silesian University of Technology, B. Krzywoustego 4, 44-100 Gliwice, Poland

[b] TCM Group, Cavendish Laboratory, University of Cambridge, 19 JJ Thomson Avenue, Cambridge CB3 0HE, United Kingdom

[c] CIC nanoGUNE, Tolosa Hiribidea 76, 20018 Donostia-San Sebastián, Spain

[d] Ikerbasque, Basque Foundation for Science, 48013 Bilbao, Spain

[e] Monash University, School of Chemistry, Clayton, VIC 3800, Australia

*Corresponding authors: bogumila.kumanek@polsl.pl; karolina.milowska@gmail.com; dawid.janas@polsl.pl

‡ These authors contributed equally to this work

Materials and Methods

1. The method of doping of SWCNTs with nitrogen compounds

The names and structures of all the compounds used in this study to dope SWCNTs are illustrated below. Each compound was added to acetone at a concentration of 0.1 M and then the resulting solution was used for dipping SWCNT films.

Amines

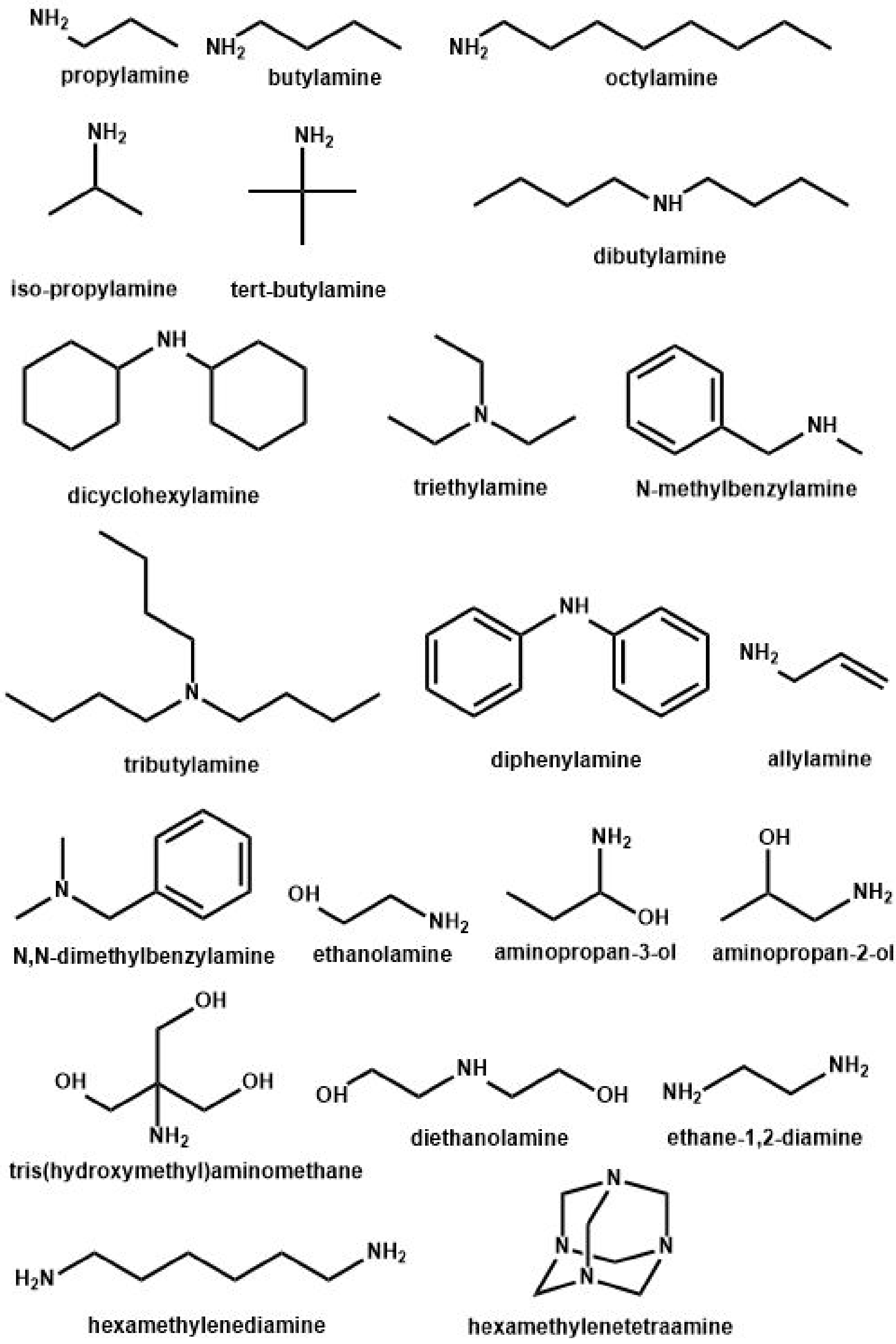

Anilines

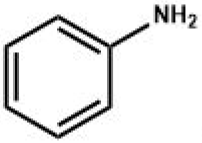

aniline

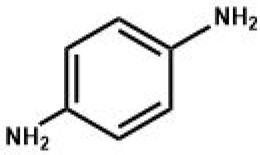

p-phenylenediamine

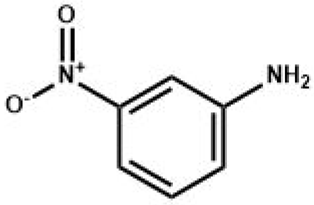

3-nitroaniline

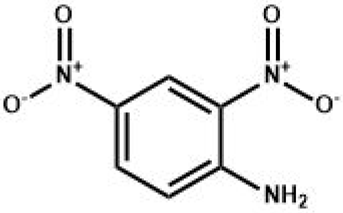

2,4-dinitroaniline

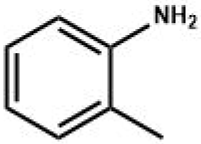

2-methylaniline

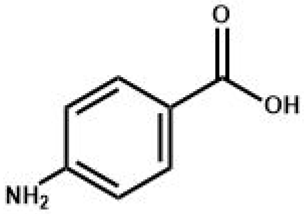

4-aminobenzoic acid

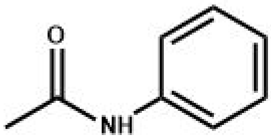

acetanilide

Pyridines

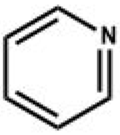

pyridine

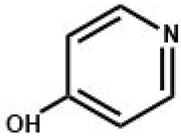

4-hydroxypyridine

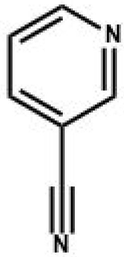

3-cyanopyridine

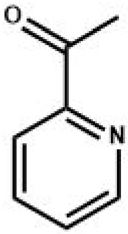

2-acetylpyridine

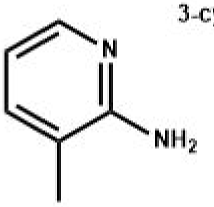

2-amino-3-methylpyridine

Azoles

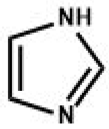

imidazole

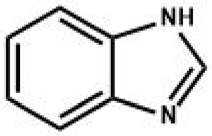

benzimidazole

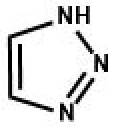

triazole

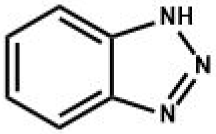

benzotriazole

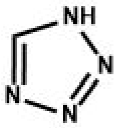

tetrazole

Pyrazine

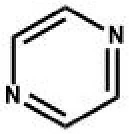

2. Modeling

DFT structural and electronic calculations of (5,5) and (10,0) SWCNTs

Spin-polarized density functional theory (DFT) calculations of narrow metallic (5,5) and semiconducting (10,0) SWCNTs were performed in the generalised gradient approximation (GGA) using Perdew-Burke-Ernzerhof (PBE) parametrisation of exchange-correlation functional [S1] and double-ζ plus polarization numerical basis (DZP) sets of orbitals localized on atoms, as implemented in QunatumATK package [S2, S3]. Long-range interactions were included in the total binding energy using the methodology proposed by Grimme [S4]. The Brillouin zone was sampled using (1 × 1 × 11) k-points, while the density mesh cutoff for real-space integrals was set to 300 Ry. During all calculations, the self-consistent field (SCF) cycle was iterated until the density matrix by less than 10^{-6} per iteration. The geometry optimization was performed until a maximum force converged to lower than 0.005 eV/Å and and the maximum stress changed by less than 0.1 GPa.

DFTB electron transport calculations of (12,12) and (20,0) SWCNTs

The thermoelectric properties of SWNTs of roughly 1.6 nm in diameter have been studied in the DFTB-NEGF approach with Slater–Koster parameterization for C, N and H atoms (auorg-1-1) as implemented in QuantumATK [S2,S5-S7]. Metallic (12,12) and semiconducting (20,0) were treated as two-probe systems with the central scattering region containing the nitrogen compound coupled to a fully relaxed pure SWCNT positioned between semi-infinite electrodes, as shown in Fig. S12-14, top panels. The Brillouin zone of the two-probe system was sampled using (7x7x201) k-points. Transmission spectra were calculated using an increased 7x7 k-point sampling over the [-3,3] eV range using 1201 points.

In contrast to the DFT-NEGF calculations of SWCNTs coupled to metallic electrodes, DFTB-NEGF calculations of SWCNTs connected to pristine SWCNT electrodes of the same type of SWCNTs did not reproduce the experimental trends.

DFTB-MD simulations of SWCNT+oA and SWCNT+oB systems

Molecular dynamic (MD) simulations of (5,5), (10,0), (12,12) and (20,0) SWCNTs doped with octylamine in the A and B configurations were performed in the NVT ensemble as implemented in QuantumATK [S2,S5,S7]. Random initial velocities of all atoms were assigned according to the Maxwell-Boltzmann distribution. The relaxation time of the Nosé–Hoover thermostat was set to 2 fs. The system was annealed from 303K to 373K. The simulations were carried out with a time-step of 0.5 fs over a time period of 4 ps (8000 steps).

Supplementary Figures and Tables

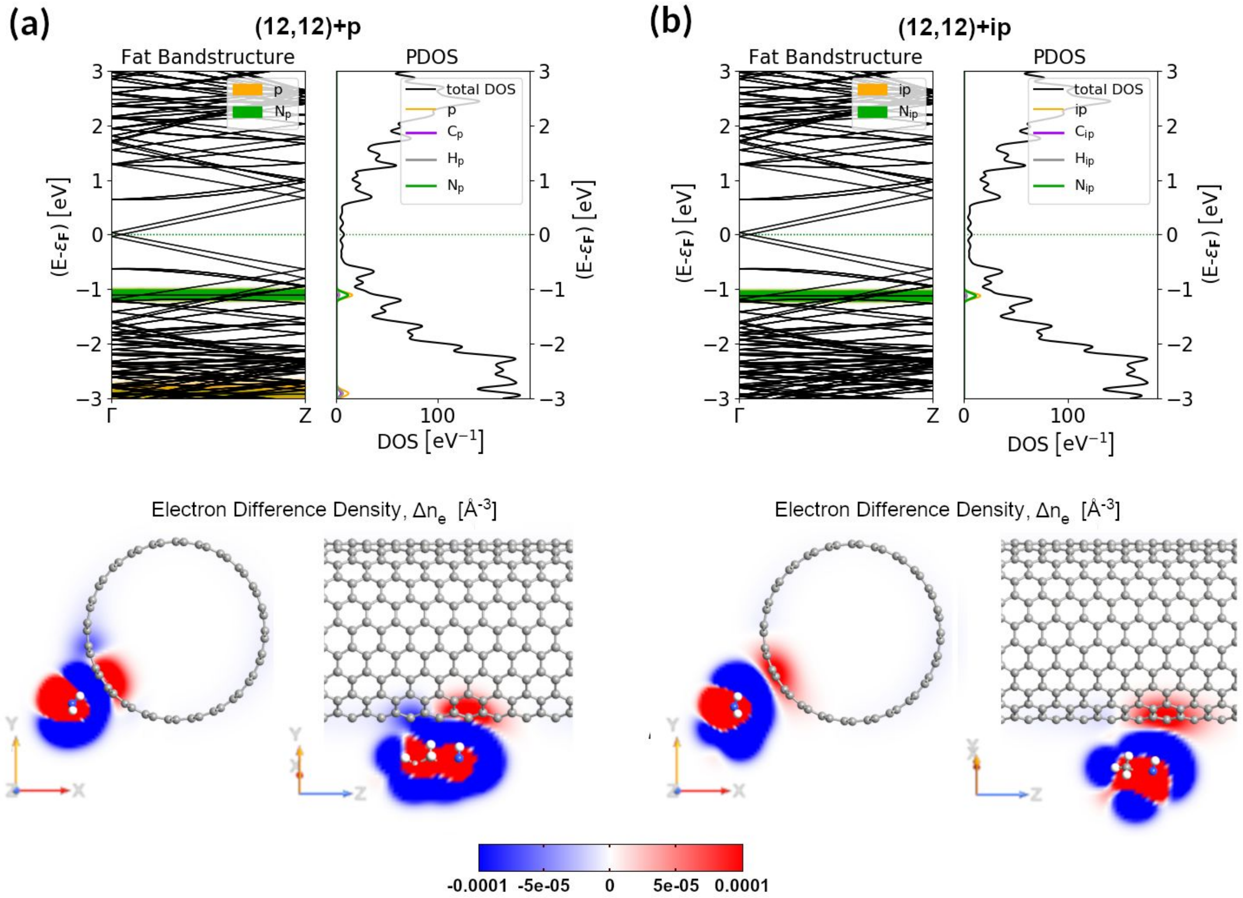

Figure S1 Computed electronic properties of (5,5) SWCNT doped with (a) propylamine (p) and (b) isopropylamine (ip) obtained with DFTB levels. (top) The fat band structures along Γ → Z of the Brillouin zone, the projected density of states on N atoms, C atoms, H atoms of the dopant species (PDOS) (bottom) and the electron difference densities. The electron difference density maps show the difference between the self-consistent valence charge density and a superposition of atomic valence densities. N and C atoms are depicted in blue and grey while H atoms are shown in white. The blue regions indicate deficiency of electrons while red regions excess of electrons. Branching in the aliphatic amines increases the optimal distance between amine and CNT (see Tab.S1) and in consequence reduces the charge transfer between the molecule and CNT (cf. electron difference densities around CNT carbon atoms in a and b panels). Also, a smaller number of additional impurity states are visible in the presented window for isopropylamine than for propylamine.

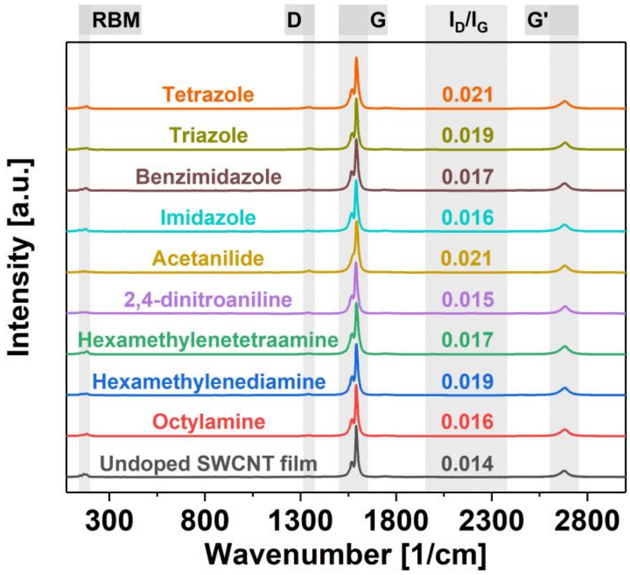

Figure S2 Raman spectra of SWCNT films doped with the most promising nitrogen-containing compounds.

The pristine SWCNT film shows very good thermal stability up to about 484°C, above which it starts to undergo thermal degradation with the maximum rate at T_{max}=681°C. It is an exceptional thermal stability for SWCNTs [S8] which can be explained by the high degree of structural perfection of the material.

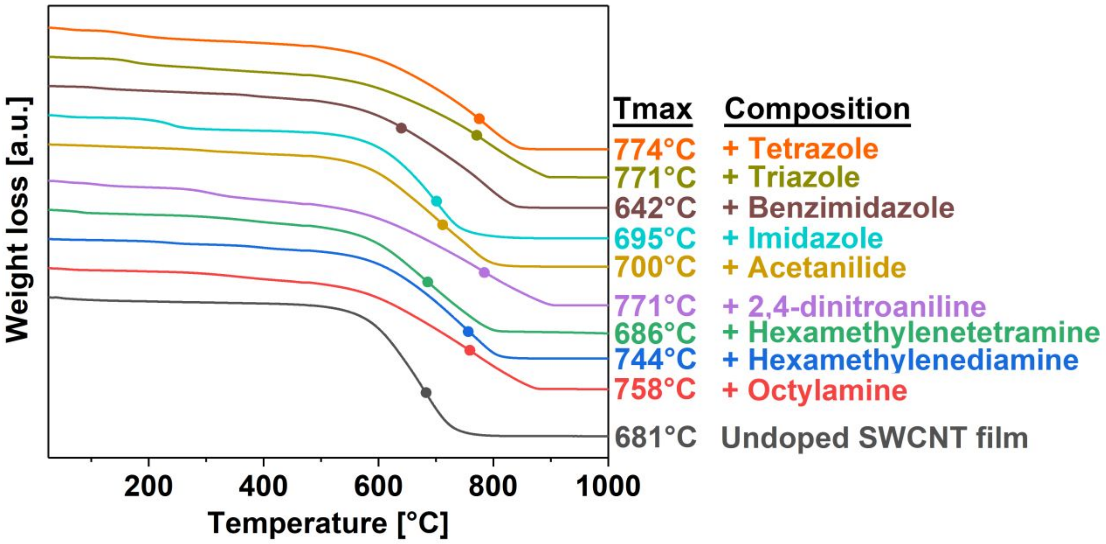

Figure S3 Thermograms of neat SWCNT film and SWCNT films after doping with nitrogen compounds. Maximum rates of degradation determined from derivative weight curves are indicated with dots.

Example thermograms for the pristine SWCNT film (Fig. S4) and the SWCNT film doped with imidazole (Fig. S5).

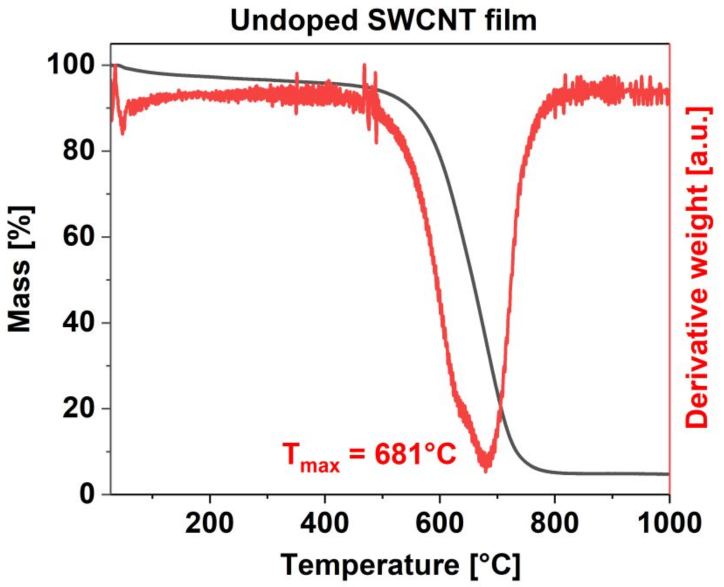

Figure S4 TGA for sample of undoped SWCNT film with the corresponding derivative weight curve.

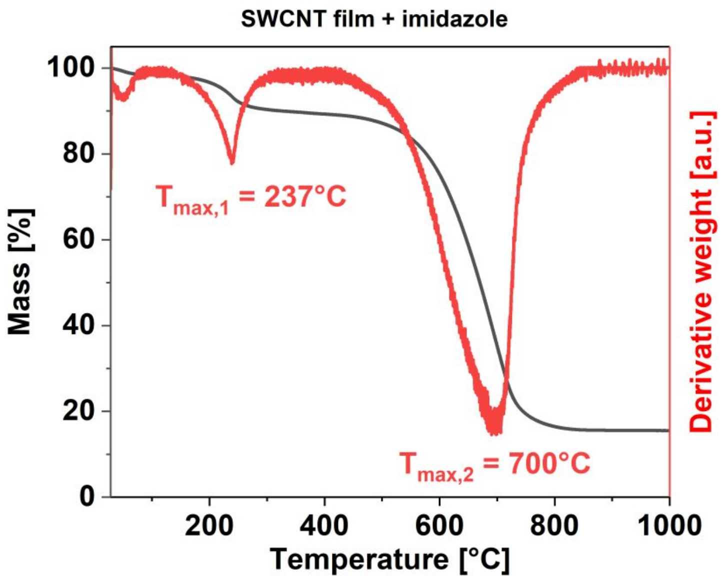

Figure S5 TGA for sample of SWCNT film doped with **i**midazole with the corresponding derivative weight curve.

Table S1 DFTB computed structural parameters, adsorption energies per nanotube carbon atom ($E_{ads/NC}$), binding energies per atom ($E_{bind/N}$), energy band gaps (E_{gap}), Fermi levels (E_F), the valence band maximum (VBM) and conduction band minimum (CBM) with respect to the Fermi levels of fully optimized pristine and nitrogen compound doped (12,12) and (20,0) SWCNTs. The nitrogen compound-SWCNT distance is the smallest difference between the molecule nitrogen or carbon distances from SWCNT symmetry axis and the mean of the SWCNT radius (see Fig. S14, top panel). The coefficient of SWCNT radius variation, CV, is defined as the ratio of the standard deviation to the mean of the SWCNT radius [S9]. The adsorption energy per carbon atom, $E_{ads/NC}$, is calculated as the difference between the total energies of fully optimized systems containing functionalized SWCNT and a nitrogen compound and the total energies of isolated SWCNT and nitrogen compound divided by the number of nanotube carbon atoms. The binding energy per atom, $E_{bind/N}$, is defined as the difference between the total energies of fully optimized systems (pure or functionalized SWCNTs or nitrogen compounds) and the sum of total atomic energies of the free atom of each type that are present in the systems, divided by the number of all atoms in the system.

SWCNT	Molecule	wt%	Nitrogen compound-SWCNT distance [Å]	CV	$E_{ads/NC}$ [eV]	$E_{bind/N}$ [eV]	E_{gap} [eV]	E_F [eV]	VBM & CBM [eV]
(12,12)	----	----	----	0.0000	----	-7.782	0	-4.6388	0
	i	1.30	3.233	0.0004	-0.00012	-7.810	0	-4.6394	0
	i-d_1	0.90	2.867	0.0009	-0.00014	-7.829	0.0025	-4.6382	-0.0014<br>0.0008
	i-d_2	0.90	3.657	0.0002	-0.00015	-7.829	0.0023	-4.6387	-0.0013<br>0.0010
	i3	3.79	2.950	0.0002	-0.00161	-7.309	0	-4.6493	0
	oA	2.43	3.332	0.0021	-0.00016	-7.779	0	-4.6501	0
	oA-d_1	1.70	2.939	0.0027	-0.00013	-7.716	0.0018	-4.6471	-0.0010<br>0.0008
	oA-d_2	1.70	3.546	0.0003	-0.00005	-7.715	0.0022	-4.6448	-0.0012<br>0.0010
	oB	2.43	3.363	0.0025	-0.00009	-7.779	0	-4.6439	0
	oC	2.43	6.889	0.0018	-0.00007	-7.779	0	-4.461	0
	hA	2.19	3.527	0.0007	-0.00016	-7.937	0	-4.6560	0
	hB	2.19	3.443	0.0013	-0.00013	-7.932	0	-4.6241	0
	hB_3	6.30	3.336	0.0026	-0.00024	-8.036	0	-4.6149	0
	hB_7	13.55	3.425	0.0011	-0.00057	-8.193	0	-4.6248	0
	p	1.13	3.526	0.0009	-0.00003	-7.893	0	-4.6467	0
	ip	1.13	4.514	0.0021	-0.00007	-7.894	0	-4.6462	0
(20,0)	----	----	----	0.0000	----	-7.870	0.4536	-4.6309	-0.2269<br>0.2267
	i	1.40	3.165	0.0004	-0.00007	-7.803	0.4540	-4.6268	-0.2270<br>0.2270
	oA	2.62	3.313	0.0028	-0.00009	-7.802	0.4535	-4.6429	-0.2268<br>0.2267
	oB	2.62	3.310	0.0023	-0.00003	-7.802	0.4542	-4.6367	-0.2271<br>0.2271
	oC	2.62	6.923	0.0025	7e-07	-7.802	0.4544	-4.6333	-0.2273<br>0.2271
	hA	2.36	3.499	0.0013	-0.00008	-7.491	0.4385	-4.6486	-0.2269<br>0.2269
	hB	2.36	3.524	0.0014	-0.00005	-7.491	0.4542	-4.6231	-0.2272<br>0.2270

Table S2 Global and local maxima of decomposition rates for SWCNT films doped with nitrogen compounds. Corresponding boiling points (b.p.) are also given in the table.

Sample name	Major T_{max} value [°C]	Minor T_{max} values [°C]	b.p. of the nitrogen compound [°C]
Undoped SWCNT film	681	-	-
+ Octylamine	758	357<br>642	176
+ Hexamethylenediamine	744	238<br>394	204
+ Hexamethylenetetramine	686	82<br>366	280<br>(sublimation)
+ 2,4-Dinitroaniline	771	108<br>309<br>659	Decomposes
+ Acetanilide	700	299<br>748	304
+ Imidazole	695	237	256
+ Benzimidazole	642	356<br>758	360
+ Triazole	771	171	203<br>(1,2,3-triazole)<br>260<br>(1,2,4-triazole)
+ Tetrazole	774	160	220

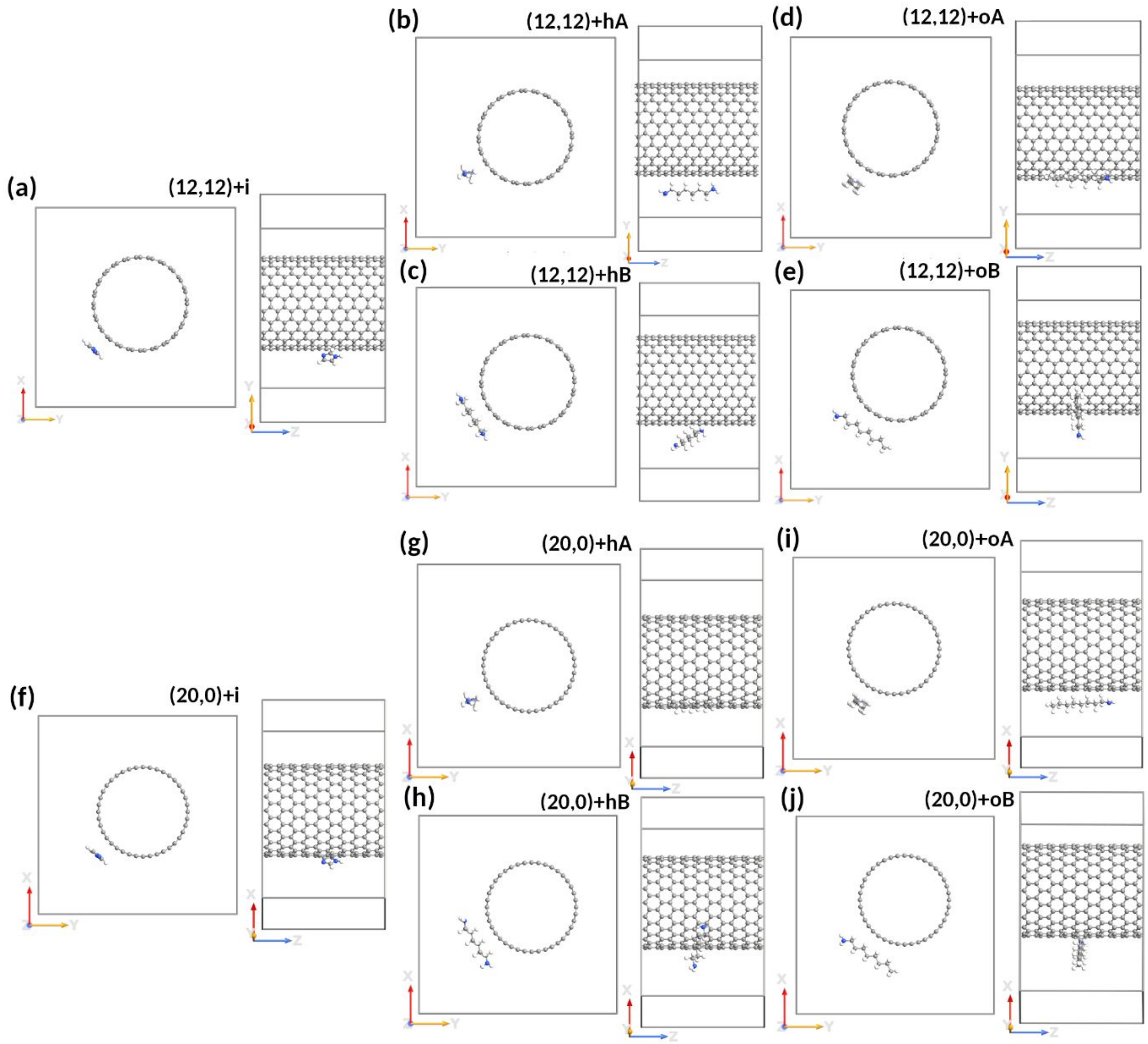

Figure S6 Atomistic cross-sectional and side views of fully optimized (a-e) (12,12) and (f-j) (20,0) SWCNTs interacting with imidazole (i), hexamethylenediamine (h) and octylamine (o). Supercells are marked by grey lines. C atoms are depicted in grey, while H and N atoms are shown in white and blue, respectively.

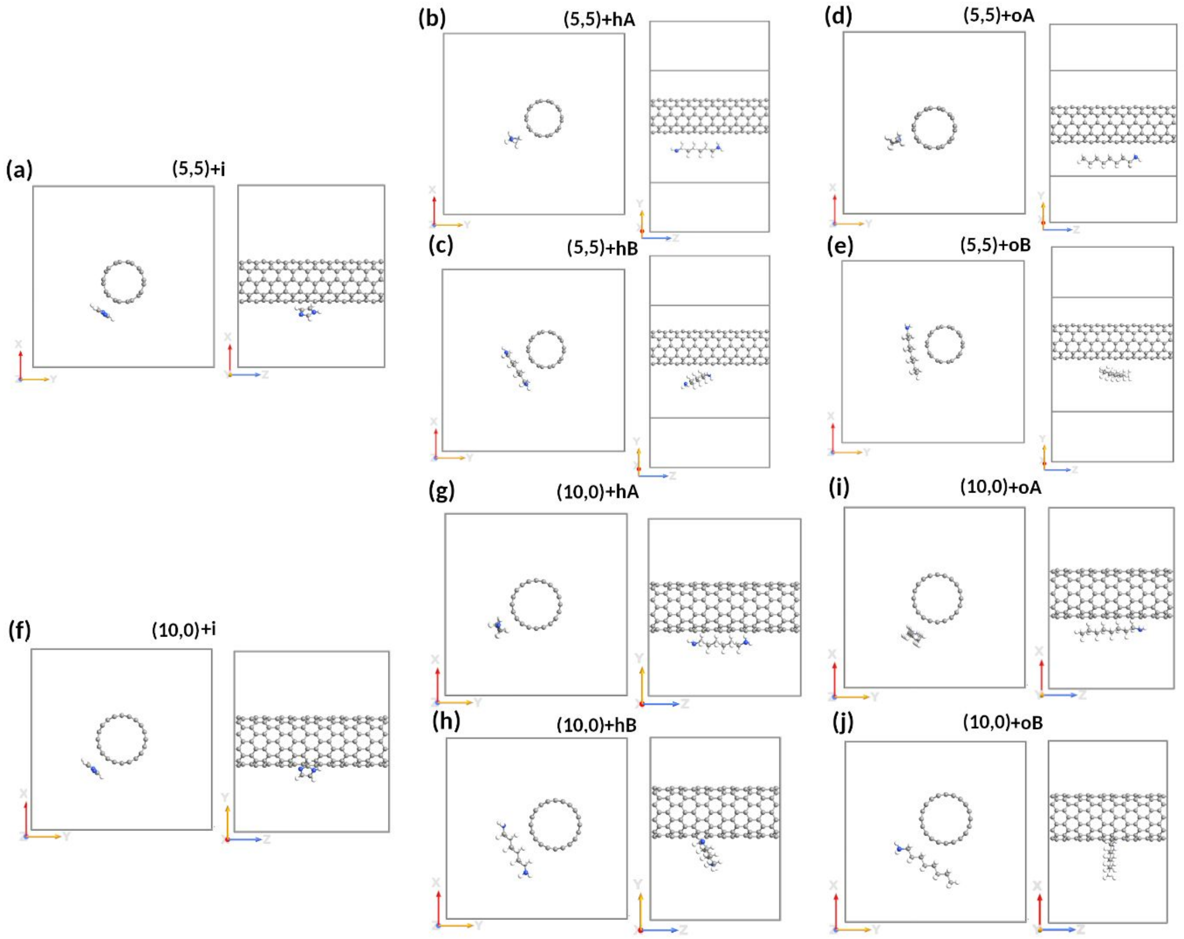

Figure S7 Atomistic cross-sectional and side views of fully optimized (5,5) and (10,0) SWCNTs interacting with imidazole (i), hexamethylenediamine (h) and octylamine (o). Supercells are marked by grey lines. C atoms are depicted in grey, while H and N atoms are shown in white and blue, respectively.

Table S3 DFTB computed structural parameters, adsorption energies per carbon atom ($E_{ads/NC}$), binding energies per atom ($E_{bind/N}$), energy band gaps (E_{gap}), Fermi levels (E_F), valence band maximum (VBM) and conduction band minimum (CBM) of fully optimized pristine and nitrogen compound doped (5,5) and (10,0) SWCNTs.

SWCNT	Molecule	wt%	Nitrogen compound-SWCNT distance [Å]	CV	$E_{ads/NC}$ [eV]	$E_{bind/N}$ [eV]	E_{gap} [eV]	E_F [eV]	VBM & CBM [eV]
(5,5)	----	----	----	0.0000	----	-7.730	0	-4.4966	0
	i	2.76	2.990	0.0003	-0.00013	-7.606	0	-4.4951	0
	oA	5.11	3.217	0.0002	-0.00009	-7.349	0	-4.4981	0
	oB	5.11	3.324	0.0003	-0.00009	-7.349	0	-4.5072	0
	hA	4.61	3.450	0.0004	-0.00025	-7.301	0	-4.5283	0
	hB	4.61	3.392	0.0004	-0.00015	-7.301	0	-4.4644	0
(10,0)	----	----	----	0.0000	----	-7.774	0.7503	-4.5807	-0.3753<br>0.3750
	i	2.76	3.144	0.0002	-0.00065	-7.649	0.7374	-4.5700	-0.3688<br>0.3686
	oA	5.11	3.371	0.0003	-0.00073	-7.340	0.7373	-4.6001	-0.3686<br>0.3687
	oB	5.11	3.431	0.0001	-0.00057	-7.340	0.7382	-4.5876	-0.3692<br>0.3690
	hA	4.61	3.448	0.0004	-0.00076	-7.389	0.7371	-4.6140	-0.3689<br>0.3682
	hB	4.61	3.534	0.0003	-0.00064	-7.388	0.7380	-4.5642	-0.3692<br>0.3688

Table S4 DFT computed structural parameters, adsorption energies per carbon atom ($E_{ads/NC}$), binding energies per atom ($E_{bind/N}$), energy band gaps (E_{gap}), Fermi levels (E_F), valence band maximum (VBM) and conduction band minimum (CBM) of fully optimized pristine and nitrogen compound doped (5,5) and (10,0) SWCNTs.

SWCNT	Molecule	wt%	Nitrogen compound-SWCNT distance [Å]	CV	$E_{ads/NC}$ [eV]	$E_{bind/N}$ [eV]	E_{gap} [eV]	E_F [eV]	VBM & CBM [eV]
(5,5)	----	----	----	0.0001	----	-8.162	0	-3.7665	0
	i	2.76	2.825	0.0007	-0.00602	-8.028	0.0022	-3.7709	-0.0010<br>0.0012
	oA	5.11	2.876	0.0063	-0.00778	-7.699	0.0019	-3.7830	-0.0007<br>0.0011
	oB	5.11	3.201	0.0081	-0.00662	-7.699	0.0014	-3.8455	-0.0006<br>0.0008
	hA	4.61	3.124	0.0057	-0.00796	-7.750	0.0054	-3.8478	-0.0024<br>0.0028
	hB	4.61	2.809	0.0057	-0.00778	-7.749	0.0047	-3.7138	-0.0022<br>0.0025
(10,0)	----	----	----	0.0000	----	-8.205	0.7857	-3.8773	-0.3915<br>0.3942
	i	2.76	2.860	0.0009	-0.00488	-8.091	0.7814	-3.8742	-0.3904<br>0.3910
	oA	5.11	3.186	0.0011	-0.00878	-7.808	0.7778	-3.9501	-0.3869<br>0.3910
	oB	5.11	3.068	0.0020	-0.00476	-7.806	0.7819	-3.9225	-0.3897<br>0.3921
	hA	4.61	3.138	0.0009	-0.00828	-7.852	0.7777	-3.9589	-0.3879<br>0.3898
	hB	4.61	2.846	0.0031	-0.00794	-7.852	0.7793	-3.8411	-0.3880<br>0.3913

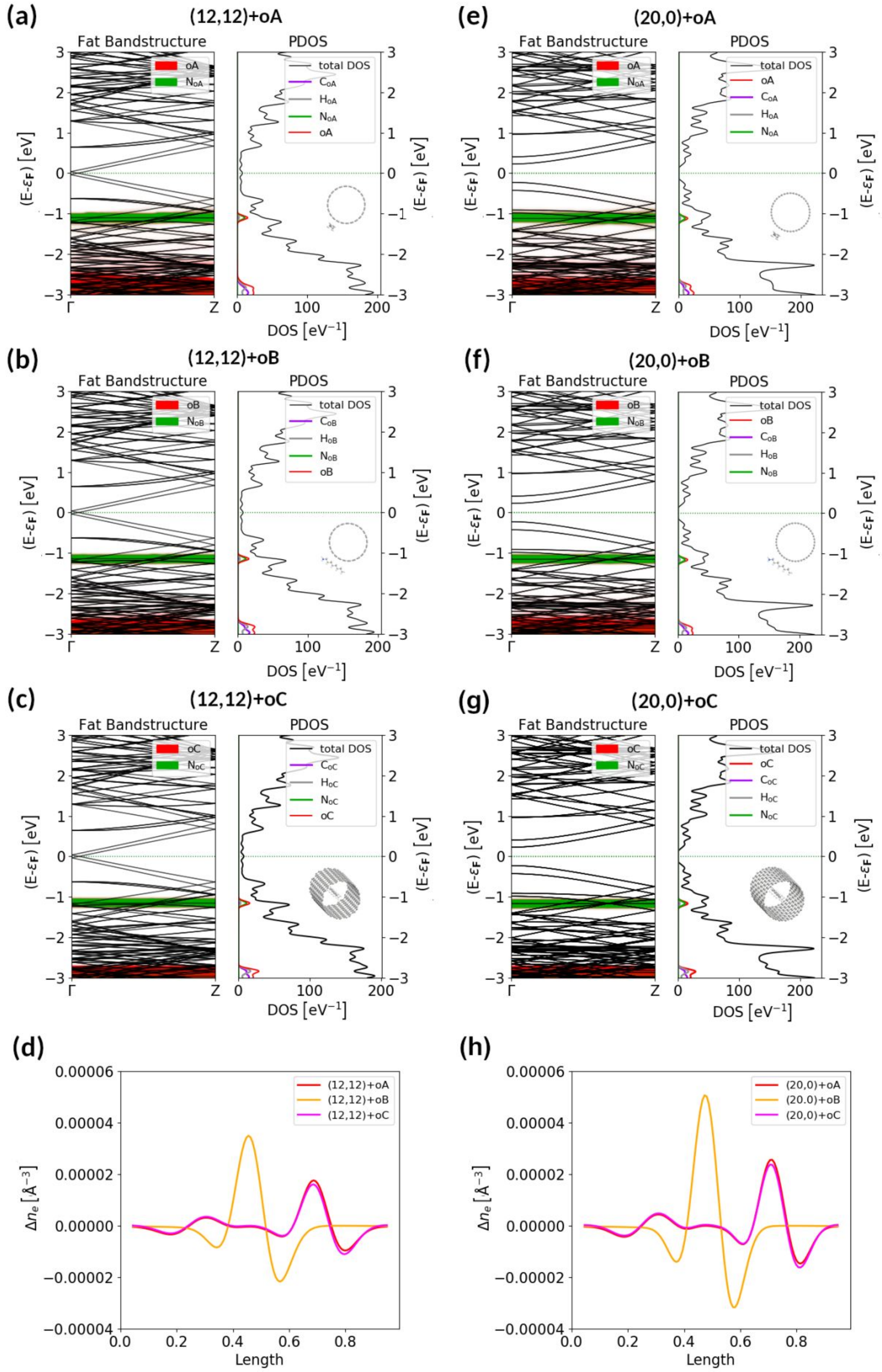

Figure S8 DFTB computed electronic properties of (a,b,c) (12,12) and (e,f,g) (20,0) SWCNTs doped with octylamine oriented along (oA) and perpendicular (oB) to the SWCNT symmetry axis and located inside the SWCNT (oC). Insets: Atomistic cross-sectional views of fully optimized systems. (a-c & e-g) The fat band structures along Γ → Z of the Brillouin zone together with the projected density of states on octylamine, amine N, amine C, and amine H (PDOS). (d,h) The macroscopic averages of 1D projections of electron difference densities on the z-axis (along SWCNT symmetry axis) for all three octylamine orientations.

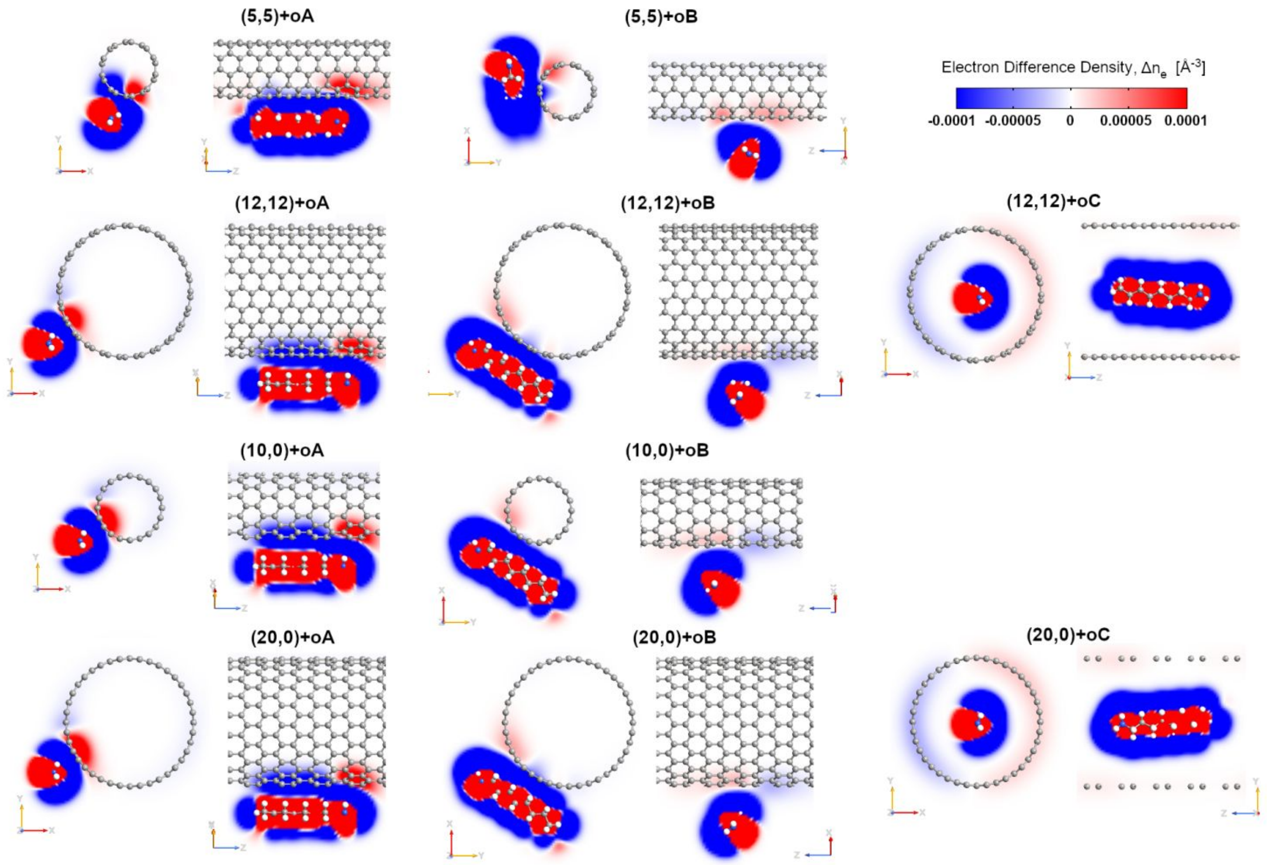

Figure S9 Electron difference density (EDD) maps of fully optimized (5,5), (12,12), (10,0) and (20,0) SWCNTs doped with octylamine oriented (left) along, (center) perpendicular to the CNT symmetry axis and (right) located inside the SWCNTs. The electron difference density maps show the difference between the self-consistent valence charge density and a superposition of atomic valence densities. The cut-planes pass through the N atom. N and C atoms are depicted in blue and grey while H atoms are shown in white. The blue regions indicate a deficiency of electrons while red regions an excess of electrons. Surprisingly, the overall charge transfers induced by octylamine in the oC configuration for both types of nanotubes are more similar to these induced by octylamine in the oB configuration rather than in the oA configuration. While the blue regions are clearly larger than the red regions around the nanotube carbon atoms in the SWCNT+oA systems, in the SWCNT+oB and SWCNT+oC systems the ratio of the red and blue regions completely changes.

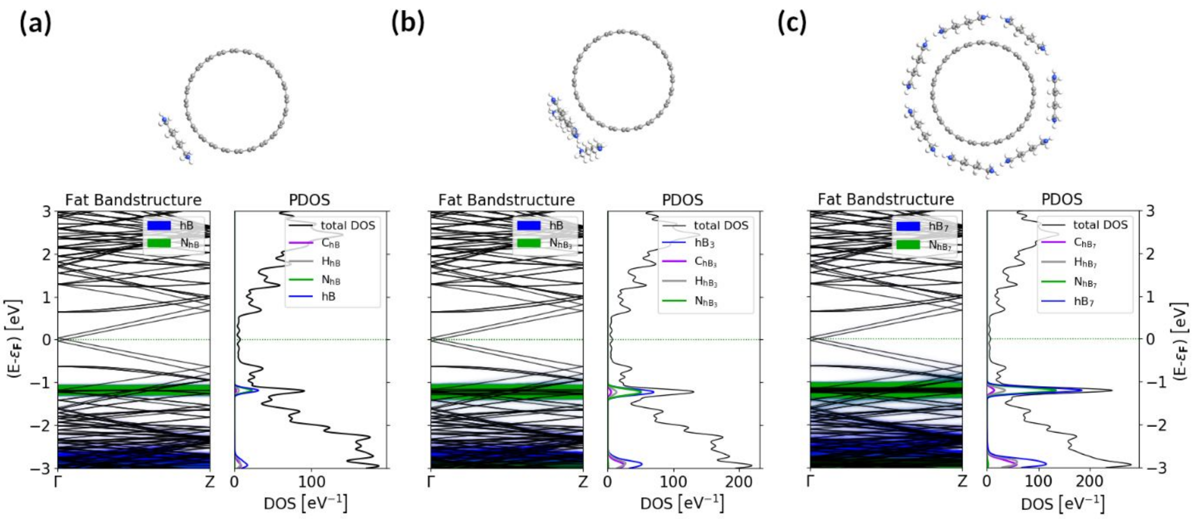

Figure S10 DFTB computed electronic properties of (12,12) SWCNTs doped with different concentration of hexamethylenediamine (h) – (a) 1 hexamethylene molecule per SWCNT, (b) 2 hexamethylene molecules per SWCNT, and (c) 7 hexamethylene molecules per SWCNT. The fat band structures along Γ → Z of the Brillouin zone together with the projected density of states on hexamethylenediamine, amine N, amine C, and amine H (PDOS). Atomistic cross-sectional views of fully optimized systems are presented above.

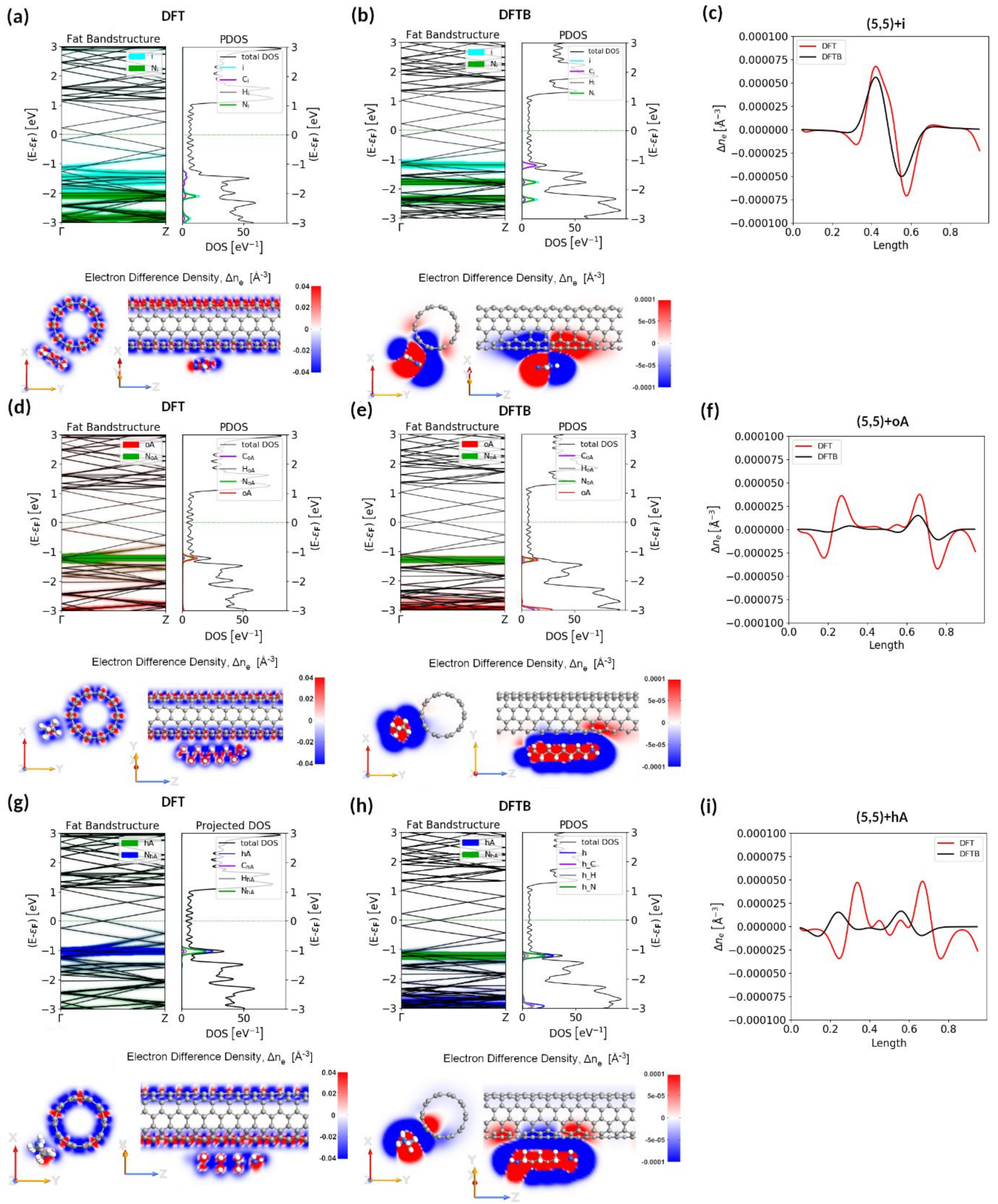

Figure S11 Computed electronic properties of (5,5) SWCNT doped with (a-c) imidazole, (d-f) octylamine and (g-i) hexamethylenediamine obtained at the DFT and DFTB levels. (a,b,d,e) The fat band structures along Γ → Z of the Brillouin zone, the projected density of states on molecule N, molecule C, and molecule H species (PDOS) (top) and the electron difference densities (bottom). The electron difference density maps show the difference between the self-consistent valence charge density and the superposition of atomic valence densities. N and C atoms are depicted in blue and grey while H atoms are shown in white. The blue regions indicate deficiency of electrons while red regions excess of electrons. (c,f,i) Comparison between DFT and DFTB calculated macroscopic averages of 1D projections of electron difference densities on the z-axis (along SWCNT symmetry axis) for (c) imidazole, (f) octylamine and (i) hexamethylenediamine doped (5,5) CNT. The macroscopic average was taken in range of [0.1, 0.9] of the primitive lattice vector along z-direction and plotted using Gaussian kernel with a variance given by the window length.

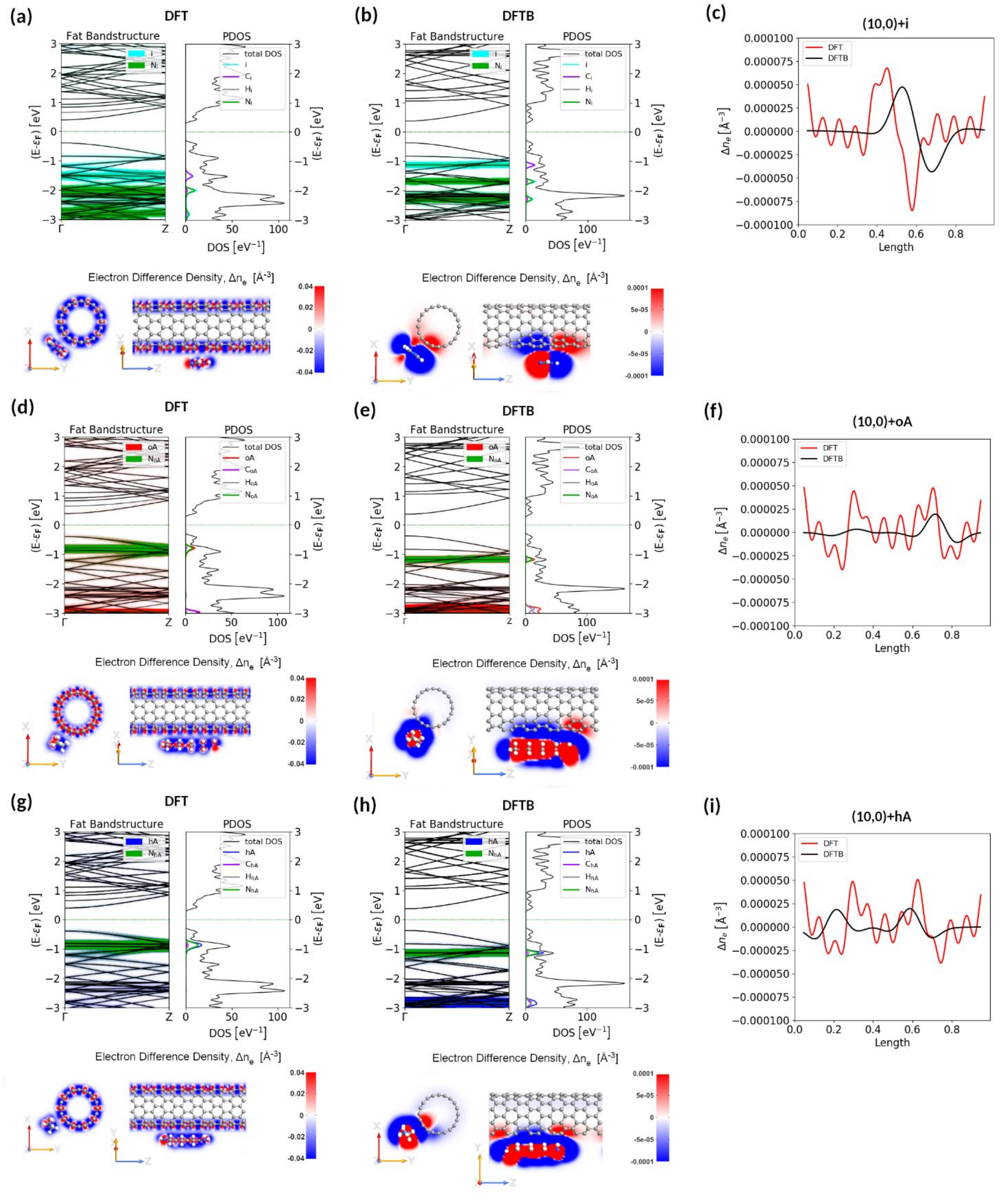

Figure S12 Computed electronic properties of (10,0) SWCNT doped with (a-c) imidazole, (d-f) octylamine and (g-i) hexamethylenediamine obtained at the DFT and DFTB levels.

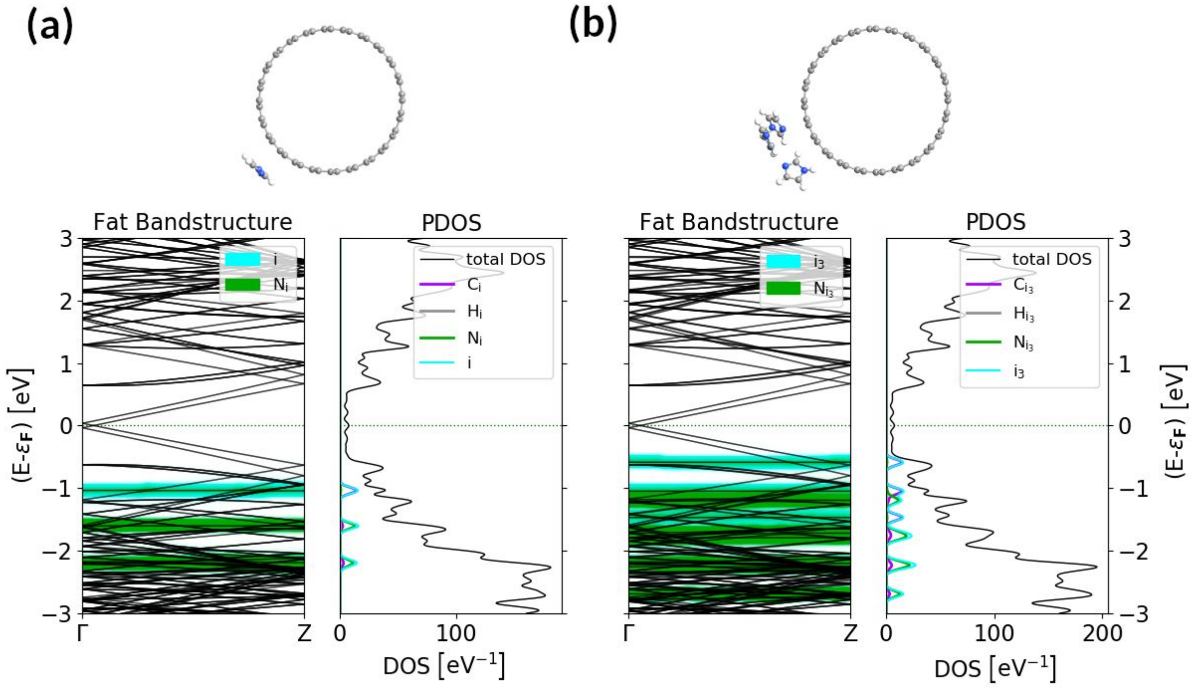

Figure S13 DFTB computed electronic properties of (12,12) SWCNTs doped with different concentration of imidazole (i) – (a) 1 imidazole molecule per SWCNT, and (b) 3 imidazole molecules per SWCNT. The fat band structures along Γ → Z of the Brillouin zone together with the projected density of states on imidazole, azole N, azole C, and azole H (PDOS). Atomistic cross-sectional views of fully optimized systems are presented above.

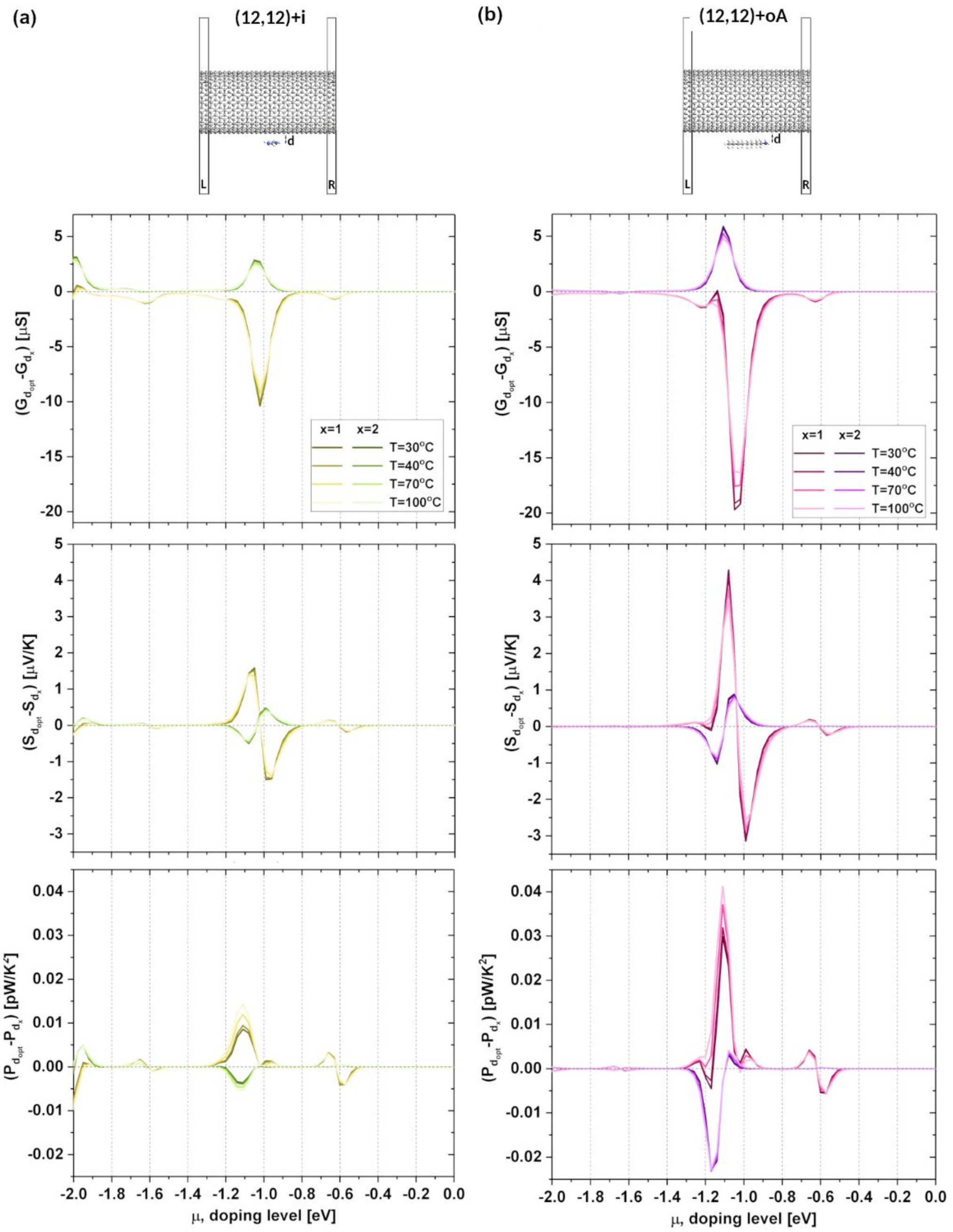

Figure S14 DFTB computed thermoelectric properties of (12,12) SWCNT doped with (a) imidazole and (b) octylamine placed closer (d_1) and further (d_2) from the SWCNT symmetry axis than lowest energy configuration. Changes in electrical conductance (G), Seebeck coefficient (S) and power factor per nanotube (P) with respect to the fully optimized systems are plotted as a function of doping level (μ) for two different nitrogen compound-SWCNT distances. For convenience, only p-doping region is displayed. The atomistic side view of the models used for the transport calculation with marked nitrogen compound-SWCNT distance, d, are presented above. The semi-infinite electrodes consisting of perfect SWCNTs are marked by grey frames at the top.

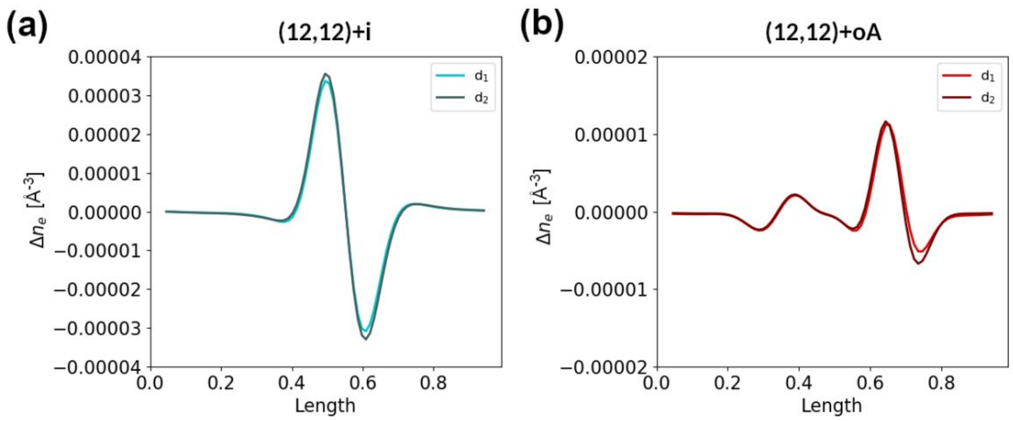

Figure S15 Macroscopic averages of 1D projections of electron difference densities of (a) imidazole and (b) octylamine doped (12,12) SWCNT on the z-axis (along SWCNT symmetry axis computed at DFTB level. The macroscopic average was taken in range of [0.1, 0.9] of the fractional z-coordinate and plotted using Gaussian kernel with a variance given by the window length. Nitrogen compounds around the lateral surface of the SWCNT were placed at two different distances from the nanotube ($d_1<d_2$). Systems were fully optimized but the position of one of the molecule nitrogen atoms as well as positions of most distant two neighbouring rows of SWCNT carbon atoms along z-direction were kept fixed.

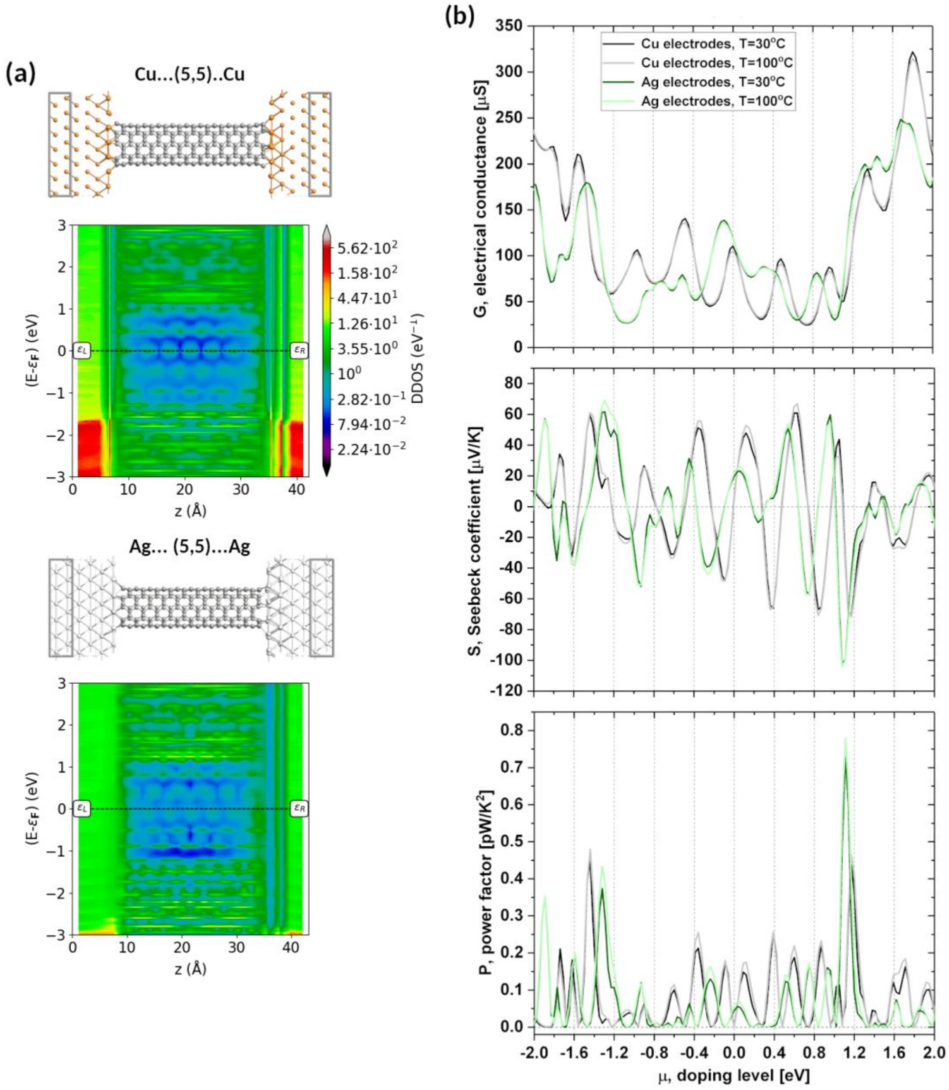

Figure S16 DFT computed electronic and thermoelectric properties of metallic (5,5) SWCNT coupled to Ag and Cu electrodes. (a) The energy resolved local device density of states (LDDOS) and side views of both systems. (b) The electrical conductance (G), Seebeck coefficient (S) and Power Factor per nanotube (P) are plotted as a function of doping level (μ) for both types of electrodes.

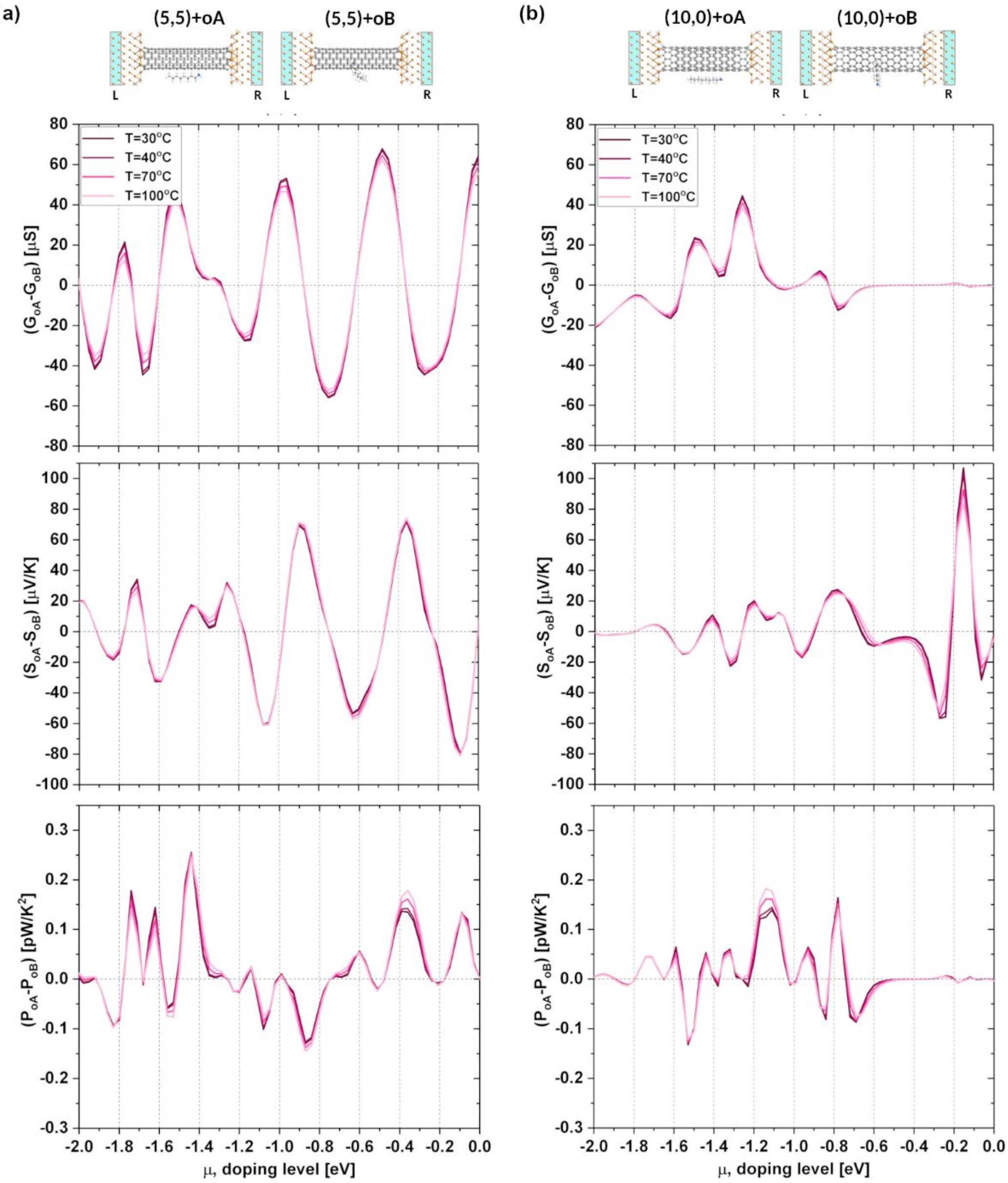

Figure S17 DFT computed thermoelectric properties of (a) metallic (5,5) and (b) semiconducting (10,0) SWCNT doped with octylamine oriented along (oA) and perpendicular (oB) to SWCNT symmetry axis. Differences in electrical conductance (G), Seebeck coefficient (S) and Power Factor per nanotube (P) between both orientations are plotted as a function of doping level (μ) for three different nitrogen compound-SWCNT distances. For convenience, only p-doping region is displayed. The atomistic side view of fully optimized models used for the transport calculations are shown above. The semi-infinite Cu electrodes are marked are highlighted in blue.

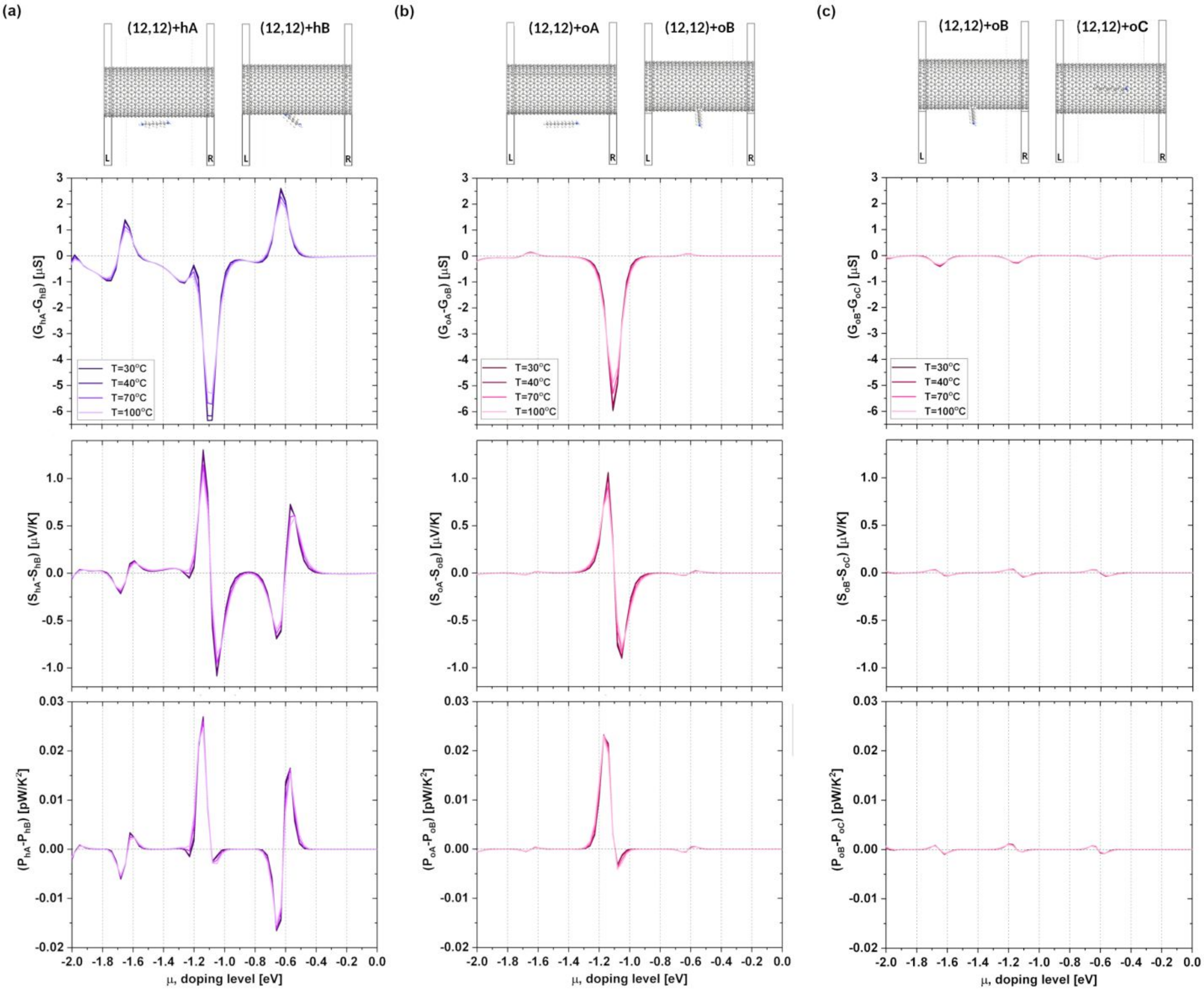

Figure S18 DFTB computed thermoelectric properties of (12,12) SWCNT doped with (a) hexamethylenediamine and (b, c) octylamine oriented along (hA, oA) and perpendicular (hB, oB) to the SWCNT symmetry axis and located inside SWCNT (oC). Differences in electrical conductance (G), Seebeck coefficient (S) and Power Factor per nanotube (P) between both orientations are plotted as a function of doping level (μ). For convenience, only p-doping region is displayed. The atomistic side view of fully optimized models used for the transport calculations are shown above. The semi-infinite electrodes consisting of perfect SWCNTs are marked by grey frame at the top.

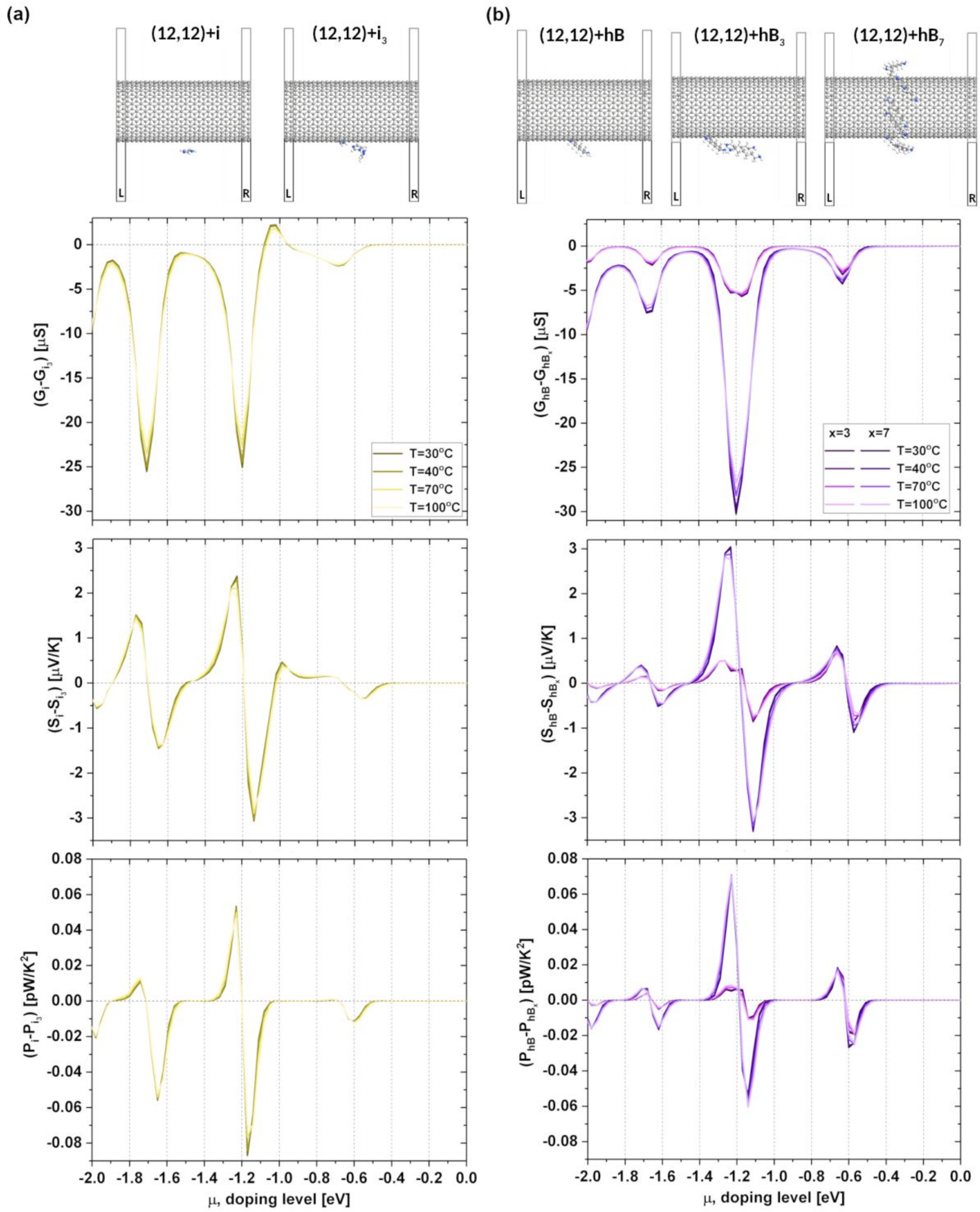

Figure S19 DFTB computed thermoelectric properties of (a) imidazole and (b) octylamine doped (12,12) SWCNT. Differences in electrical conductance (G), Seebeck coefficient (S) and Power Factor per nanotube (P) for different concentrations of both nitrogen compounds plotted as a function of doping level (μ). For convenience, only p-doping region is displayed. The atomistic side views of the models used for the transport calculations are presented above. The semi-infinite electrodes consisting of perfect SWCNTs are marked by grey frame at the top.

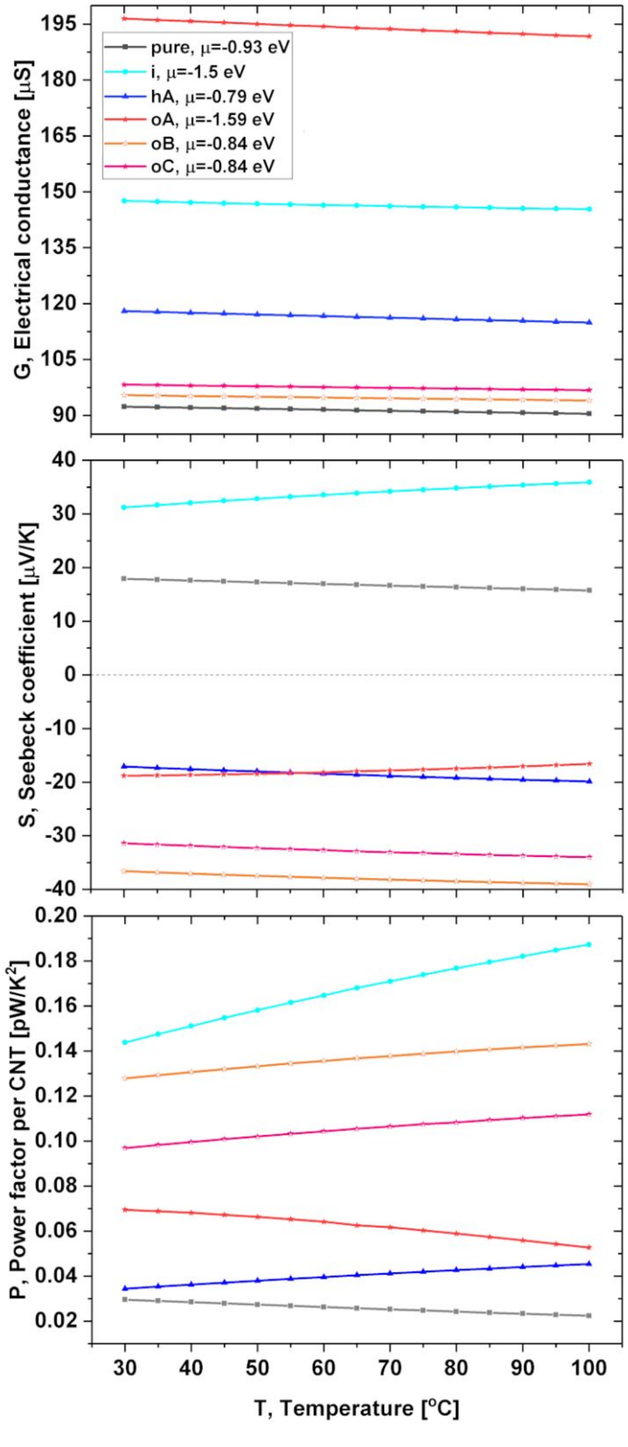

Figure S20 DFT computed thermoelectric properties of pristine and doped mixed parallel SWCNT circuits placed between metallic electrodes. G, S, P at different temperatures for pristine and imidazole (i), octylamine (o), and hexamethylenediamine (h) doped SWCNT films. All three systems containing octylamine, oriented along (oA) and perpendicular (oB) to the SWCNT symmetry axis, as well as located inside the nanotube (oC), are shown. The oC model, similarly to oB model allows to reproduce only partially the lower-temperature behavior of CNT samples observed in experiments. However, as the fabrication method of SWCNT films leaves majority of SWCNTs capped, it is unlikely that octylamine will be located inside the nanotubes.

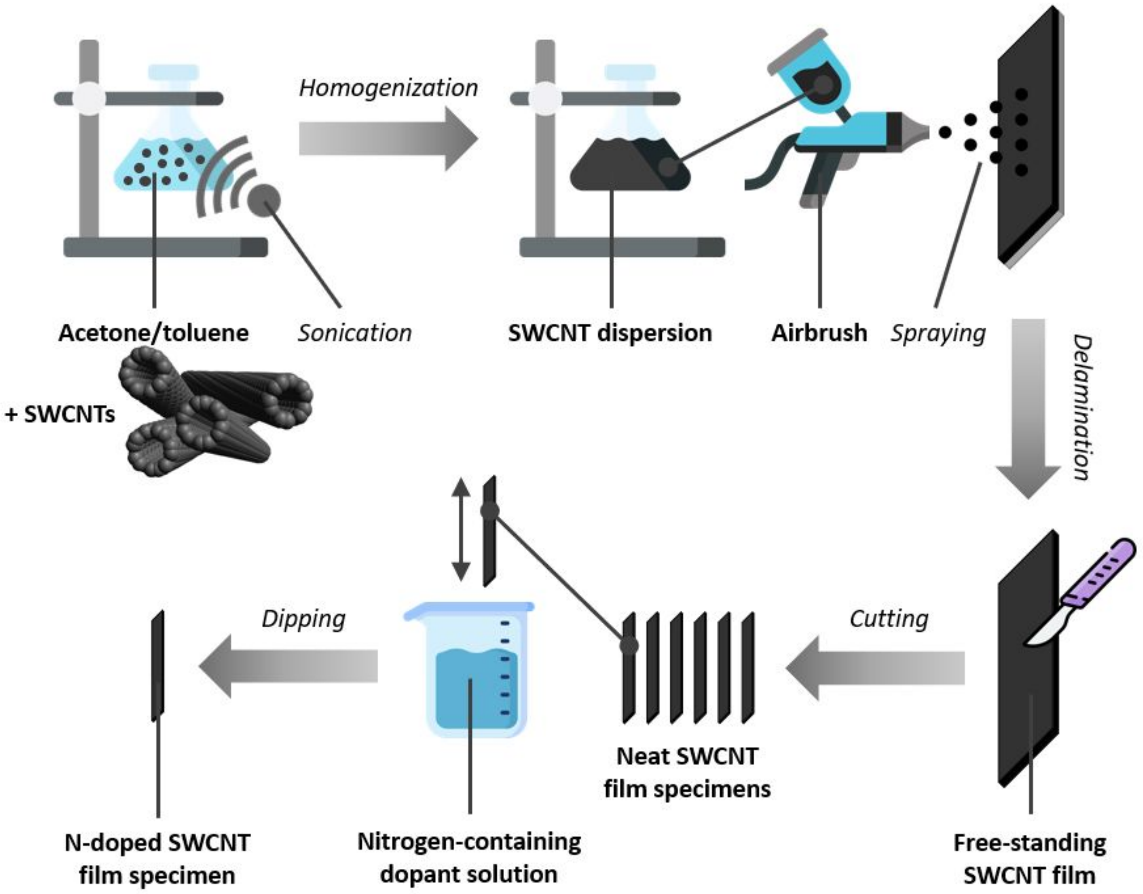

Figure S21 Preparation of free-standing films from SWCNTs by doping with nitrogen compounds. Pictograms were obtained from Freepik (www.flaticon.com) and Avogadro: an open-source molecular builder and visualization tool (www.avogadro.cc).

References

S1. Perdew, J. P., Burke, K. & Ernzerhof, M. Generalized gradient approximation made simple. *Phys. Rev. Lett.* **77**, 3865–3868 (1996).

S2. Synopsys QuantumATK version 2020.09-sp1, https://www.synopsys.com/silicon/quantumatk.html (accessed 16-07-2021.)

S3. S. Smidstrup et al., QuantumATK: an integrated platform of electronic and atomic-scale modelling tools. J. *Phys.: Condens. Matter* **32**, 015901 (2020).

S4. S. Grimme, Semiempirical GGA-type density functional constructed with a long-range dispersion correction. *J. Comput. Chem.* **27**, 1787–1799 (2006).

S5. K. Stokbro, D. E. Petersen, S. Smidstrup, A. Blom, M. Ipsen and K. Kaasbjerg, Semiempirical model for nanoscale device simulations, Phys. Rev. B **82**, 075420 (2010).

S6. Brandbyge, M.; Mozos, J.-L. ; Ordejón, P.; Taylor J.; Stokbro, K. Density-functional method for nonequilibrium electron transport, Phys. Rev. B: Condens. Matter, 65(16), 165401 (2002).

S7 A. Fihey, C. Hettich, J. Touzeau, F. Maurel, A. Perrier, C. Köhler, B. Aradi, and T. Frauenheim, SCC-DFTB parameters for simulating hybrid gold-thiolates compounds, Journal of Computational Chemistry, 36, pp. 2075-2087, 2015

S8 D. Janas, A.C. Vilatela, and K.K. Koziol, Performance of carbon nanotube wires in extreme conditions, Carbon, 62, pp. 438-446, 2013

S9 Milowska, K. Z. Influence of Carboxylation on Structural and Mechanical Properties of Carbon Nanotubes: Composite Reinforcement and Toxicity Reduction Perspectives. *J. Phys. Chem. C*, **2015**, *119*, 26734–26746.
